# Interpretable Machine Learning for Thermal Conductivity Prediction of Silica Aerogel–Incorporated Cementitious Composites

**DOI:** 10.3390/gels12080714

**Published:** 2026-08-12

**Authors:** Jingjing Zhang, Ning Liang

**Affiliations:** 1 School of Civil Engineering and Architecture, Guangxi University of Science and Technology, Liuzhou 545006, China; 20240301014@stdmail.gxust.edu.cn; 2 Guangxi Zhuang Autonomous Region Engineering Research Center of Geotechnical Disaster and Ecological Control, Guangxi University of Science and Technology, Liuzhou 545006, China; 3 Guangxi Key Laboratory of Bamboo and Timber Structure Digital–Intelligent Construction, Guangxi University of Science and Technology, Liuzhou 545006, China

**Keywords:** silica aerogel, cementitious composites, thermal conductivity, machine learning, SHAP analysis

## Abstract

Silica aerogel–incorporated cementitious composites possess low density and thermal conductivity. Their thermal conductivity is influenced by the interplay of mix composition, pore structure, mineral admixtures, and environmental testing conditions. In this study, a literature-derived database containing 208 data records was established for thermal conductivity prediction. Eight variables were utilized as inputs: aerogel content, water-to-cement ratio (W/C), sand content, foam content, silica fume content, fly ash content, testing temperature, and testing relative humidity. Thermal conductivity was designated as the output. The models developed for this study included XGBoost, random forest (RF), support vector regression (SVR), and their counterparts optimized using particle swarm optimization (PSO), which were subsequently compared. Among the optimized models, PSO–SVR showed the most balanced predictive performance, with test-set *R*^2^, RMSE, and MAE values of 0.9306, 0.1005, and 0.0658, respectively. SHAP analysis identified sand content as the most important variable, followed by W/C and aerogel content. Mechanistically, silica aerogel reduces effective thermal conductivity by introducing low-conductivity phases, weakening solid heat–transfer networks, increasing heat–flow tortuosity, and accumulating interfacial thermal resistance. This study provides a data–driven and interpretable approach for thermal conductivity prediction and low–conductivity mix design of silica aerogel–incorporated cementitious composites.

## 1. Introduction

The continuous increase in building operational energy consumption and carbon emissions has drawn considerable attention to the application of high–efficiency thermal insulation materials in low–carbon buildings and energy-saving building envelopes [1,2,3]. Cementitious materials are extensively used in construction engineering because of their cost-effectiveness, durability, and adaptability [4]. However, conventional dense cementitious materials generally contain relatively continuous solid–phase heat-transfer pathways, resulting in comparatively high thermal conductivity and limiting their ability to meet the requirements of high–performance building envelopes for low thermal conductivity and lightweight design [5,6]. Therefore, reducing the thermal conductivity of cementitious materials while maintaining their engineering suitability has become an important focus in the development of new thermal insulation materials for buildings.

Silica aerogel is characterized by low density, high porosity, and a nanoporous structure, making it an important functional component for regulating the thermal transport behavior of cementitious composites. Previous studies have shown that incorporating silica aerogel (SiO_2_ aerogel) into mortar, concrete, foamed concrete, and ultra–high–performance cementitious materials can reduce material density and thermal conductivity while enhancing thermal insulation performance [7,8,9,10]. From the perspective of heat conduction in multiphase composites, the role of SiO_2_ aerogel does not simply arise from an increase in porosity. Instead, it reduces the effective thermal conductivity of the composite by introducing low–conductivity phases, weakening continuous solid–phase heat–transfer networks, lengthening heat–flow pathways, and increasing the interfacial thermal resistance between aerogel particles and the cementitious matrix [11,12,13]. However, the thermal conductivity response of silica aerogel–incorporated cementitious composites is not governed solely by aerogel content; rather, it is jointly regulated by the water–to–cement ratio, sand content, foam content, mineral admixtures, curing age, testing temperature, and testing relative humidity [14], exhibiting pronounced multifactorial coupling and nonlinear characteristics. Existing studies have mainly focused on experimental testing, mix–design optimization, and theoretical model development. Experimental methods can reveal the effects of aerogel content, porosity, testing environmental temperature and humidity, and curing age on thermal conductivity. However, they are time–consuming and costly, making it difficult to cover a wide range of mix proportions and environmental conditions. Conventional theoretical models, such as the Maxwell–Eucken model, Hamilton–Crosser model, multiphase composite models, and multiscale physical models [15,16,17,18], can explain heat–transfer behavior from the perspectives of material composition and pore structure. Nevertheless, the morphology of aerogel particles, interfacial transition zones, pore distribution, moisture state, and hydration process involve considerable uncertainty, which limits the generalization ability of traditional empirical equations and theoretical models when applied to multi–source experimental datasets.

Machine learning provides a data-driven approach for performance prediction and variable–contribution analysis of complex cementitious composites. Different machine learning algorithms have distinct learning mechanisms and applicability. Random forest (RF) improves prediction stability through bootstrap sampling and ensemble averaging, making it suitable for nonlinear and noisy datasets. Extreme Gradient Boosting (XGBoost) enhances predictive capability through gradient boosting and regularization, and is effective in capturing complex feature interactions. Support vector regression (SVR) uses kernel-based mapping to describe nonlinear relationships and often shows good generalization ability under limited sample conditions. In recent years, RF, Light Gradient Boosting Machine (LightGBM), XGBoost, and Shapley additive explanations (SHAP) have been widely applied to the prediction of strength and thermal properties of cementitious materials [19,20,21]. Prior research has shown that machine learning models can capture the nonlinear mapping relationships among mix-design parameters, curing conditions, material composition, and target properties. Additionally, SHAP-based techniques can identify the primary influencing factors [22,23]. However, there are still few machine learning investigations on the thermal conductivity of cementitious composites containing silica aerogel. In particular, a systematic prediction framework that simultaneously considers mix–design parameters, pore–regulating factors, mineral admixtures, curing age, and testing temperature and relative humidity is still lacking. Moreover, high prediction accuracy alone is insufficient to support material design. Interpretable analysis should be further integrated with multiphase heat–transfer mechanisms to determine whether the key variables identified by the model are physically consistent.

Based on these considerations, a literature-derived database for the thermal conductivity of silica aerogel–incorporated cementitious composites was established in this study. Eight variables, including aerogel content, W/C, sand content, foam content, silica fume content, fly ash content, testing temperature, and testing relative humidity, were used as input variables, while thermal conductivity was taken as the output variable. XGBoost, RF, SVR, and their particle swarm optimization (PSO)–enhanced versions were developed. The performance of the models was comprehensively evaluated based on the coefficient of determination (*R*^2^), root mean square error (RMSE), mean absolute error (MAE), prediction error distribution, and Taylor diagram. On this basis, key variables were identified by combining correlation analysis with SHAP analysis, and the physical rationality of the model interpretations was discussed from the perspective of multiphase heat–transfer mechanisms. This study aims to establish a thermal conductivity response modeling framework that integrates predictive accuracy and interpretability, providing a reference for thermal performance evaluation and low–thermal–conductivity mix design of silica aerogel–incorporated cementitious composites.

## 2. Results and Discussion

### 2.1. Hyperparameter Optimization of Base Models

**Figure 1:**
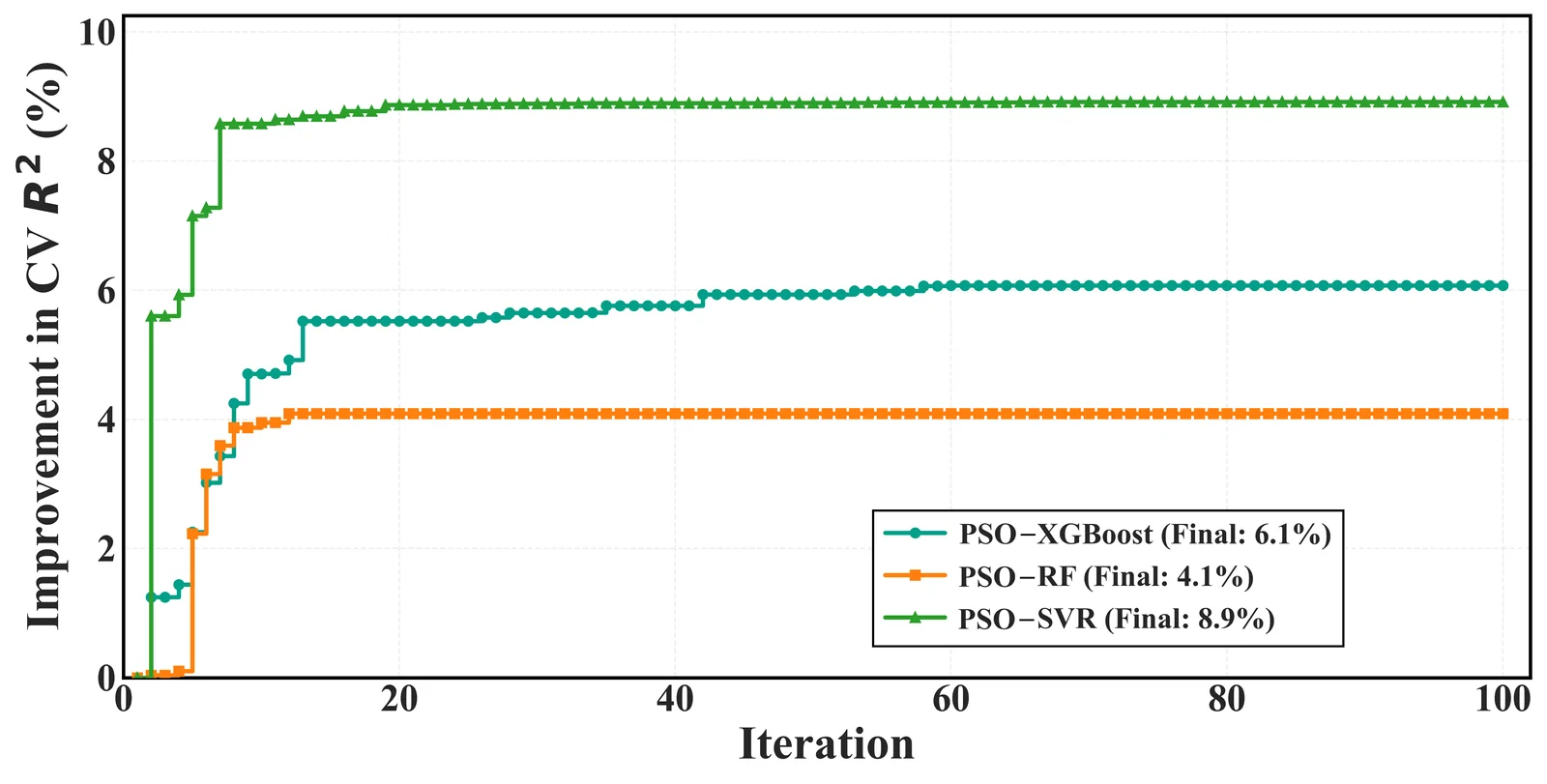
Convergence curves of the PSO–optimized models.

Particle swarm optimization (PSO) [24] was used to optimize the key hyperparameters of Extreme Gradient Boosting (XGBoost), random forest (RF), and support vector regression (SVR) to mitigate the subjectivity and uncertainty associated with empirical hyperparameter selection. The optimized hyperparameter configurations of the three PSO–optimized models are summarized in Table 1. The improvements in cross–validated *R*^2^ (CV *R*^2^) during the iterative optimization process of the three PSO–optimized models are shown in Figure 1. All three models exhibited rapid improvements during the early iterations and subsequently approached stable states. PSO–SVR showed the largest final CV *R*^2^ improvement of 8.9%, followed by PSO–XGBoost and PSO–RF with improvements of 6.1% and 4.1%, respectively. PSO–SVR increased sharply within approximately the first 10 iterations and became nearly stable after about 20 iterations, whereas PSO–RF reached a stable state relatively early. PSO–XGBoost continued to exhibit small incremental improvements until approximately 60 iterations before converging. This indicates that PSO can efficiently search the parameter space within a limited number of iterations and guide the three models from initially specified empirical hyperparameters toward a hyperparameter region associated with better generalization performance. The different convergence trajectories also indicate that the three models responded differently to hyperparameter optimization, with PSO–SVR exhibiting the greatest improvement in CV *R*^2^.

### 2.2. Comparison of Model Prediction Performance

The predictive performance of different machine learning models on both the training and test sets is presented in Table 2. All models demonstrated generally satisfactory predictive performance for thermal conductivity, although noticeable differences in prediction errors were evident between the training and test sets. Taking the test-set results as the primary evaluation criterion, XGBoost achieved the highest *R*^2^ of 0.9379 and the lowest RMSE of 0.0995, whereas PSO–XGBoost yielded the lowest MAE of 0.0561. Among the three PSO-optimized models, PSO–SVR achieved the highest *R*^2^ and the lowest RMSE, with values of 0.9306 and 0.1005, respectively. Compared with the unoptimized SVR model, PSO–SVR increased the test-set *R*^2^ from 0.8689 to 0.9306, while reducing the RMSE from 0.1446 to 0.1005 and the MAE from 0.0865 to 0.0658. This suggests that the PSO-optimized parameter combination may have enhanced the ability of SVR to characterize the multivariable nonlinear thermal conductivity response and further reduced the prediction errors.

In addition, XGBoost and RF achieved training–set *R*^2^ values of 0.9999 and 0.9807, respectively, whereas their test–set *R*^2^ values decreased to 0.9379 and 0.9110. This suggests that the base tree–based models had strong fitting capability for the local structure of the training samples, but may also have shown a certain tendency toward overfitting, thereby affecting their generalization performance on unseen samples. This behavior may originate from the high flexibility of tree-based ensemble models in capturing complex nonlinear relationships and local variations within the training dataset.

During PSO optimization, the fitness function was established based on five-fold cross-validation performance rather than training-set performance. Meanwhile, model-specific complexity-, sampling-, and regularization-related hyperparameters were optimized within predefined ranges. This strategy was designed to identify parameter combinations with improved generalization capability. After PSO optimization, the test-set performance of SVR improved consistently across all three evaluation metrics. In contrast, PSO produced mixed effects on the tree-based models. For XGBoost, the test-set *R*^2^ decreased from 0.9379 to 0.9302 and the RMSE increased from 0.0995 to 0.1055, although the MAE decreased from 0.0617 to 0.0561. For RF, the test-set *R*^2^ decreased from 0.9110 to 0.8949, while the RMSE and MAE increased from 0.1191 and 0.0672 to 0.1294 and 0.0803, respectively. These results indicate that the effect of PSO-based hyperparameter optimization was model-dependent and that improvements in cross-validation performance did not translate uniformly to the fixed test set. Among the optimized models, PSO–SVR achieved the highest test-set *R*^2^ and the lowest RMSE, whereas PSO–XGBoost achieved the lowest MAE. Therefore, SVR benefited most consistently from PSO optimization under the current multi-source database and feature combination.

**Figure 2:**
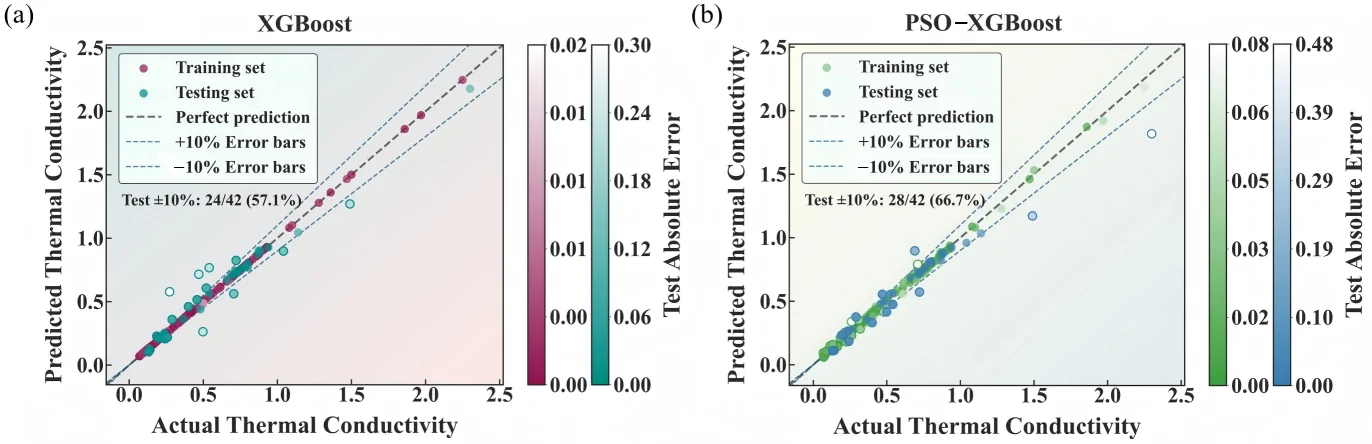

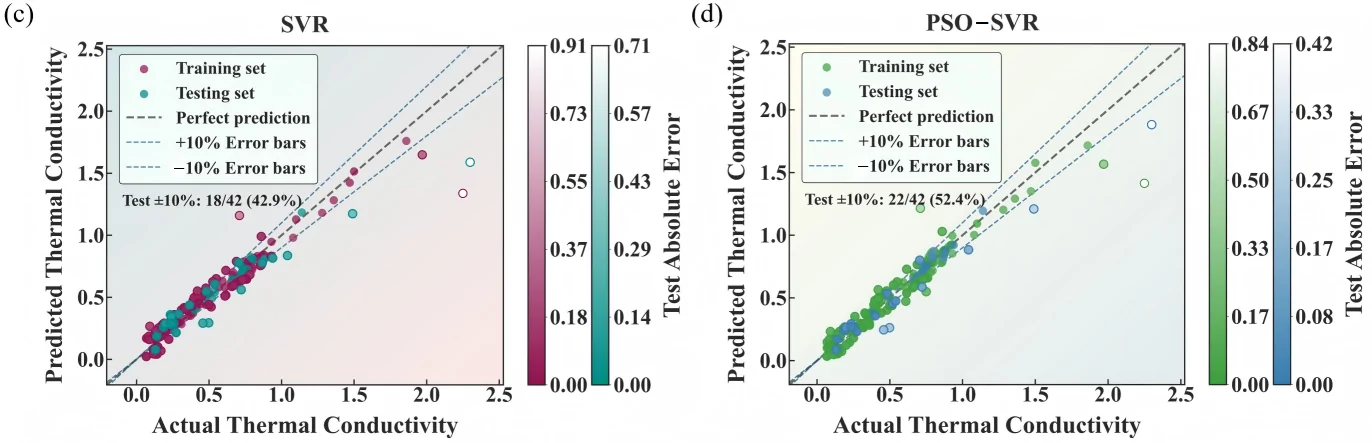

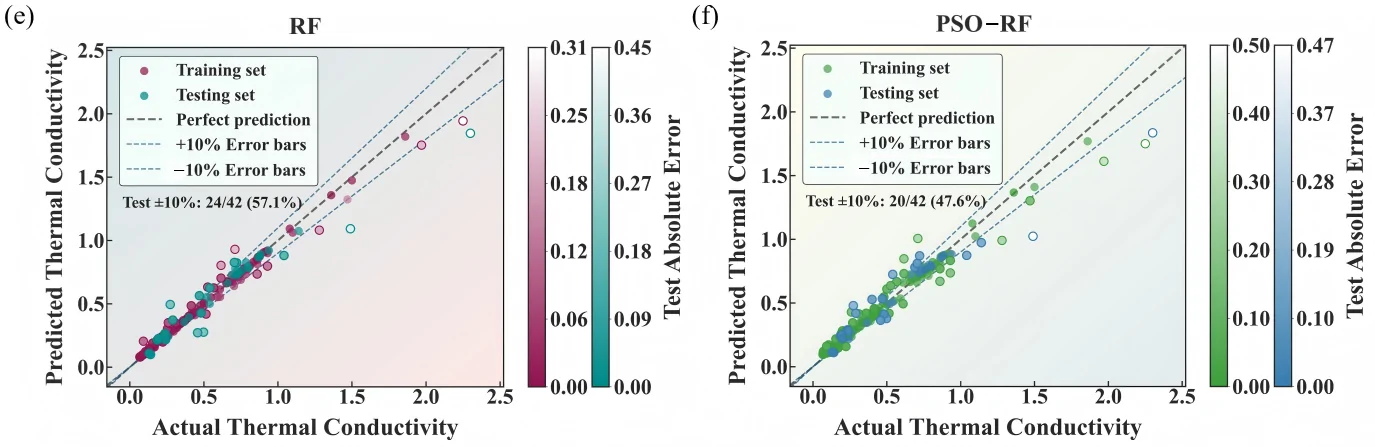
Comparison between experimental and predicted thermal conductivity values: (**a**) XGBoost; (**b**) PSO–XGBoost; (**c**) SVR; (**d**) PSO–SVR; (**e**) RF; (**f**) PSO–RF.

Figure 2 illustrates the agreement between predicted and measured values across different models. The prediction points of all models predominantly clustered close to the ideal prediction line, suggesting that the constructed machine learning models effectively captured the underlying nonlinear relationship between input variables and thermal conductivity. For the base models, the proportions of test samples falling within the ±10% error band were 57.1% for both XGBoost and RF, whereas that of SVR was 42.9%, suggesting a relatively scattered error distribution for the unoptimized SVR model. After PSO optimization, the proportion of samples within the ±10% error band increased to 52.4% for PSO–SVR, and its prediction points became more concentrated around the ideal prediction line [25]. The corresponding coverage for PSO–XGBoost also increased from 57.1% to 66.7%, whereas that for PSO–RF decreased from 57.1% to 47.6%. The changes in the error metrics were also model-dependent. Compared with XGBoost, PSO–XGBoost achieved a lower test-set MAE, although its *R*^2^ and RMSE deteriorated slightly. For PSO–RF, the test-set *R*^2^, RMSE, and MAE were all inferior to those of the corresponding base RF model. In contrast, PSO–SVR showed consistent improvements in *R*^2^, RMSE, MAE, and ±10% error-band coverage relative to the unoptimized SVR model.

Based on Table 2 and Figure 2, XGBoost achieved the highest test-set *R*^2^ and the lowest RMSE among all models, whereas PSO–XGBoost achieved the lowest MAE and the highest ±10% error-band coverage. Among the three optimized models, PSO–SVR achieved the highest test-set *R*^2^ and the lowest RMSE. These results indicate that no single model was superior across all evaluation criteria; however, SVR benefited most consistently from PSO optimization in terms of overall fitting accuracy, error magnitude, and error-band coverage.

### 2.3. Prediction and Residual Analysis

**Figure 3:**
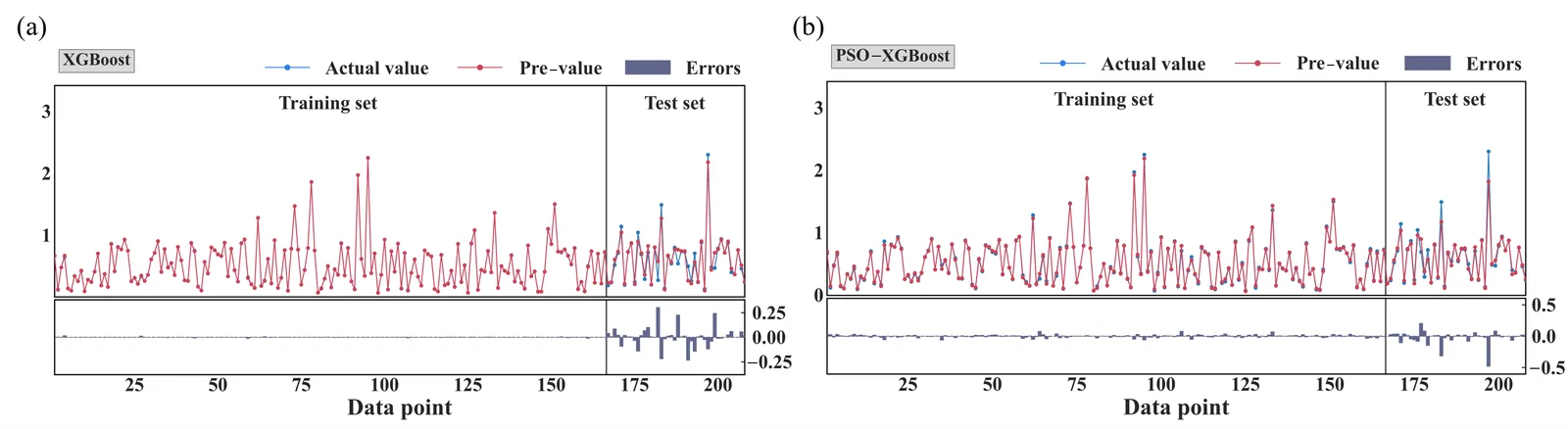

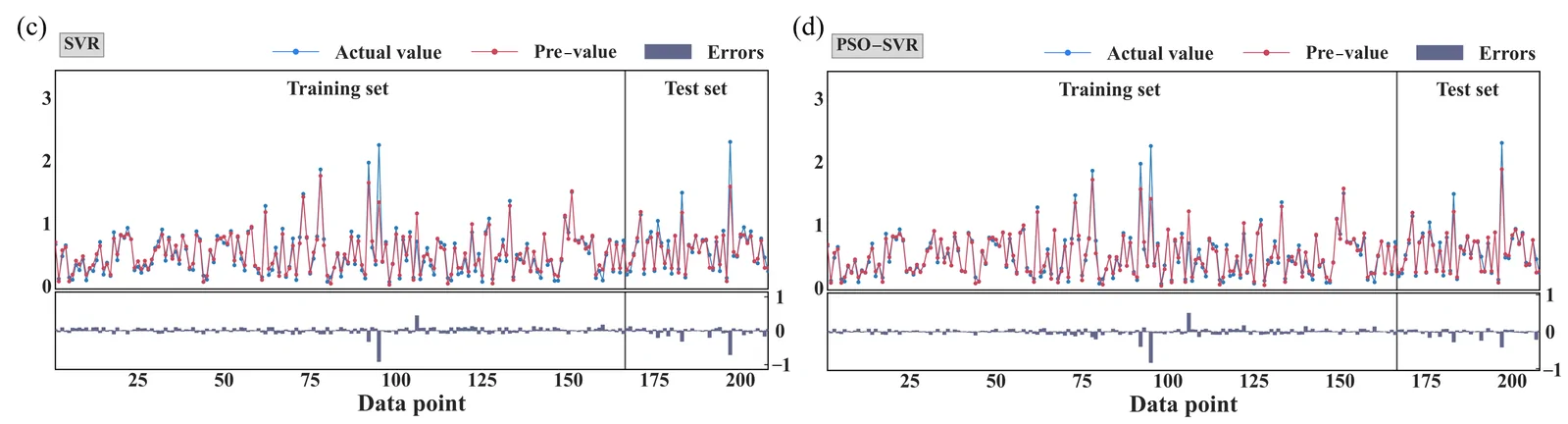

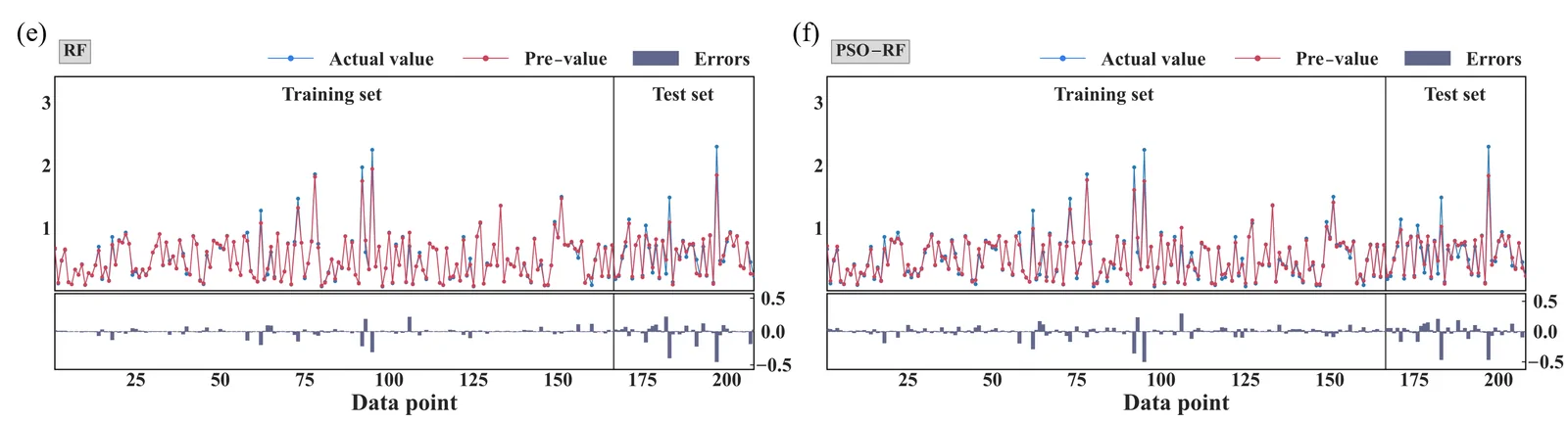
Comparison of experimental values, predicted values, and residual distributions: (**a**) XGBoost; (**b**) PSO–XGBoost; (**c**) SVR; (**d**) PSO–SVR; (**e**) RF; (**f**) PSO–RF.

The measured values, predicted values, and residual distributions of different models on the training and test sets are shown in Figure 3. The prediction curves of all models generally aligned with the variation trend of the measured thermal conductivity values. This alignment suggests that the developed machine learning models effectively captured the main nonlinear mapping between the input variables and thermal conductivity. In terms of residual distribution, XGBoost and RF exhibited relatively small errors on the training set, whereas noticeable deviations still occurred for some individual samples in the test set. This suggests that the base tree-based models had strong fitting capability for the training samples, but their generalization stability for some samples with high thermal conductivity or local fluctuations remained limited. In comparison, SVR showed a relatively scattered residual distribution, indicating that its initial parameter combination still had room for improvement in adapting to the current multivariable nonlinear thermal conductivity response. After PSO optimization, the changes in prediction–curve agreement and residual distribution were model-dependent. PSO–SVR showed improved agreement between the predicted and measured curves, and its residuals became more concentrated than those of the unoptimized SVR model. PSO–XGBoost still maintained good agreement with the measured values, although the reduction in residual amplitude was not uniform for all test samples. By contrast, PSO–RF did not show a clear reduction in test-set residual amplitudes compared with RF. Among the optimized models, PSO–SVR exhibited a relatively more concentrated residual distribution and more balanced error behavior across the training and test sets, corresponding to improved test-set generalization performance [22].

### 2.4. Comprehensive Model Evaluation

**Figure 4:**
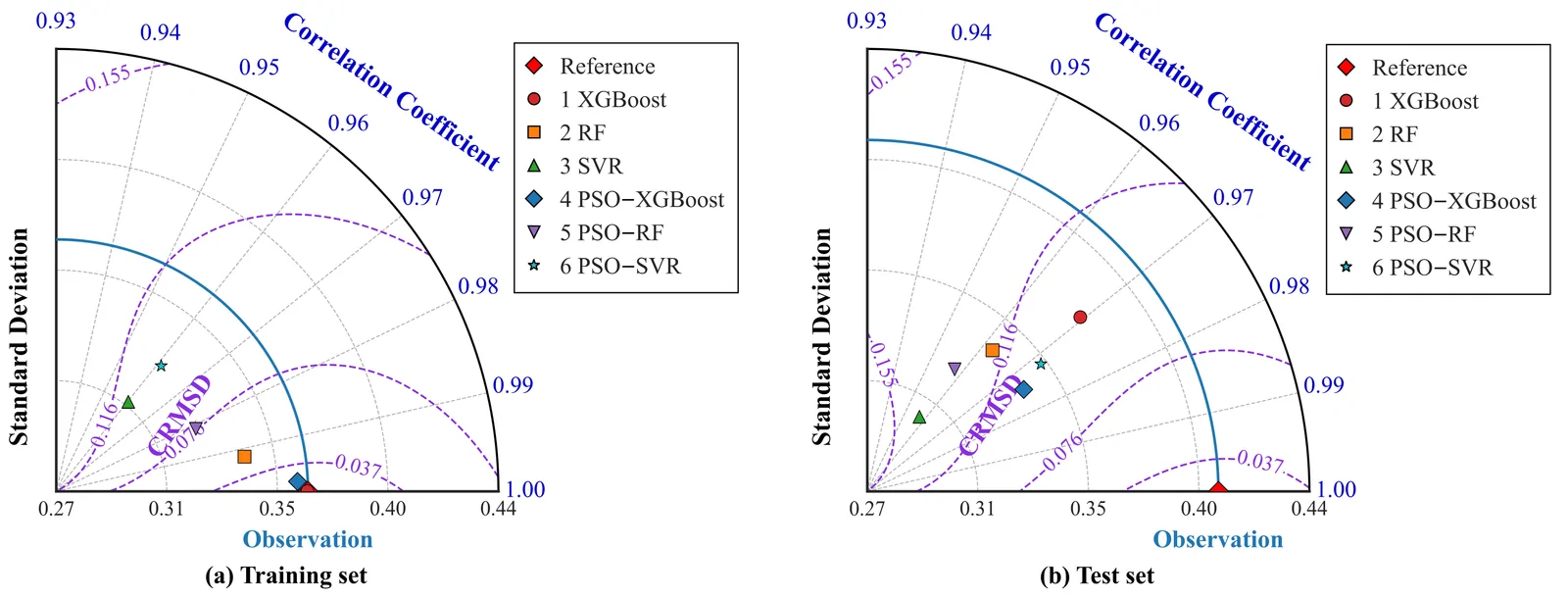
Taylor diagrams for comprehensive evaluation of model prediction performance: (**a**) Training set; (**b**) Test set.

The comprehensive predictive performance of different models on the training and test sets is illustrated in Figure 4. The Taylor diagram simultaneously presents the correlation coefficient, standard deviation, and centered root mean square error. A model point closer to the reference point indicates better agreement between the predicted and observed sequences in terms of variation trends, degrees of dispersion, and levels of error. On the training set, all models were located in the high–correlation region, indicating that the predicted sequences could effectively reconstruct the variation characteristics of the training samples. Among them, XGBoost and RF were relatively close to the reference point. However, their test–set results indicated that the fitting advantage of the base tree–based models on the training set did not fully translate into stable test–set performance. After PSO optimization, the changes in the model positions were model-dependent. PSO–SVR moved closer to the reference point than SVR, whereas PSO–RF did not show a corresponding improvement over RF, and PSO–XGBoost remained broadly comparable to XGBoost. On the test set, PSO–SVR was closest to the reference point among the PSO-optimized models and exhibited a relatively high correlation coefficient and low centered error. However, XGBoost showed a standard deviation closer to that of the measured values. This indicates that no single model was superior across all three Taylor-diagram statistics, although PSO–SVR achieved a relatively balanced comprehensive performance among the optimized models in terms of trend consistency, dispersion matching, and error control.

To further evaluate model stability, repeated random-split validation was conducted using the optimized hyperparameters. As shown in Table 3, PSO–SVR achieved the highest average *R*^2^ value and the lowest average RMSE over 20 random 80:20 splits, whereas PSO–XGBoost yielded the lowest average MAE. Moreover, PSO–SVR exhibited the smallest standard deviations for all three evaluation metrics, indicating more stable predictive performance across different data partitions. These results confirm that the favorable performance of PSO–SVR was not dependent on a single random data partition.

### 2.5. Feature Importance and SHAP Analysis

**Figure 5:**
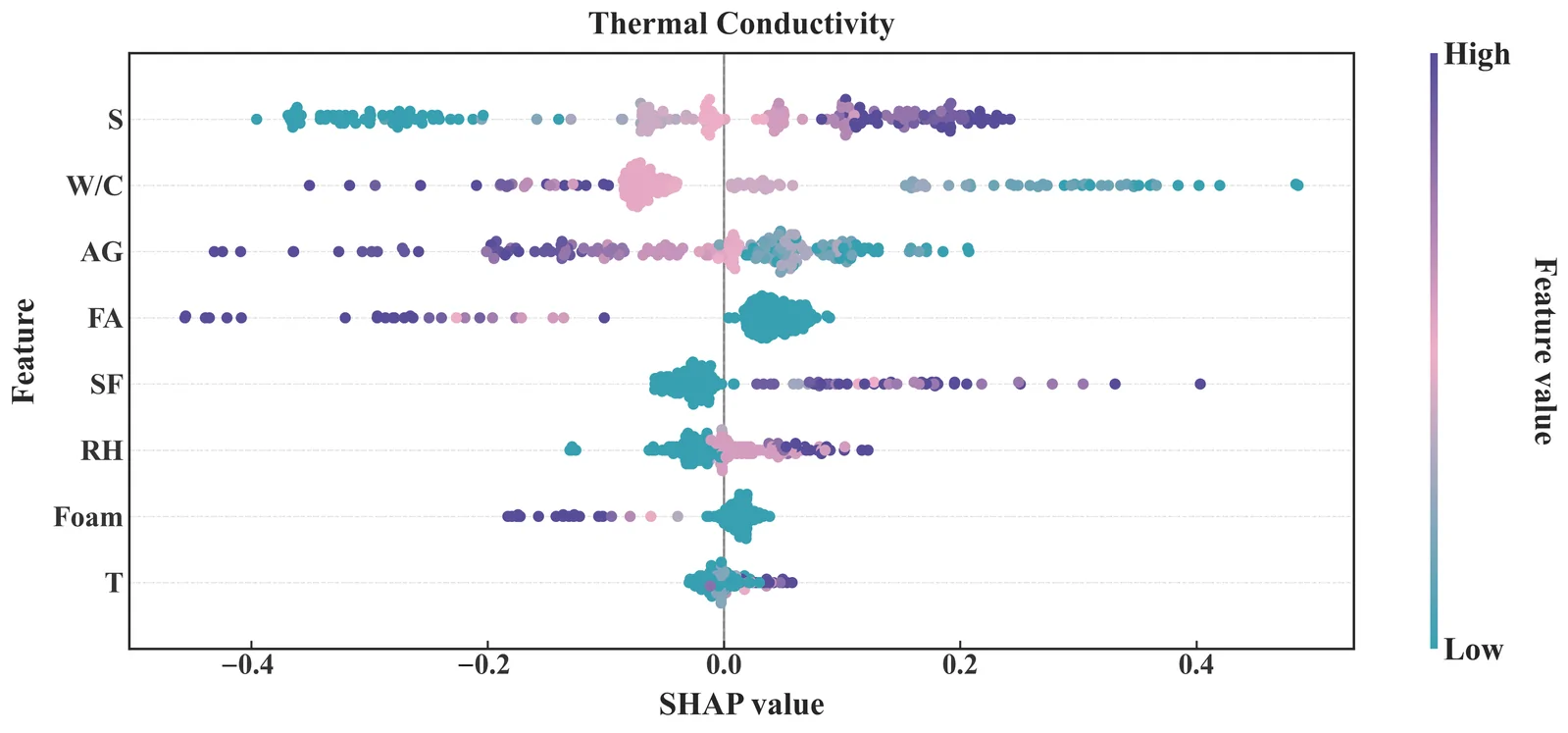
SHAP summary plot for the thermal conductivity prediction model.

The SHAP summary plot derived from the optimal PSO–SVR model is shown in Figure 5. It was used to assess the direction and magnitude of the contribution of each input variable to the predicted thermal conductivity. Sand content (S) exhibited the widest SHAP value distribution and ranked first in global importance, followed by W/C and AG, indicating that these three variables made the largest contributions to the model output. Samples with low S values were mainly associated with negative SHAP values, whereas high S values were more frequently associated with positive SHAP values, indicating that increasing sand content may enhance the continuity of the solid skeleton and increase the predicted thermal conductivity. High W/C values were predominantly associated with negative SHAP values, whereas low W/C values generally produced positive contributions, which may be related to the increased porosity and reduced matrix compactness induced by a higher W/C. Samples with high AG values were mainly associated with negative SHAP values, suggesting that, within the current data domain, higher aerogel content tended to reduce the predicted thermal conductivity. From the perspective of multiphase heat transfer, the introduction of low-conductivity aerogel phases can modify the original solid heat-transfer network and redistribute heat flow among the cementitious matrix, pore phase, and aerogel–matrix interfaces, thereby increasing heat-transfer resistance. FA, SF, and RH exhibited moderate contributions. High FA values were mainly associated with negative SHAP values, whereas high SF and RH values generally corresponded to positive SHAP values.

**Figure 6:**
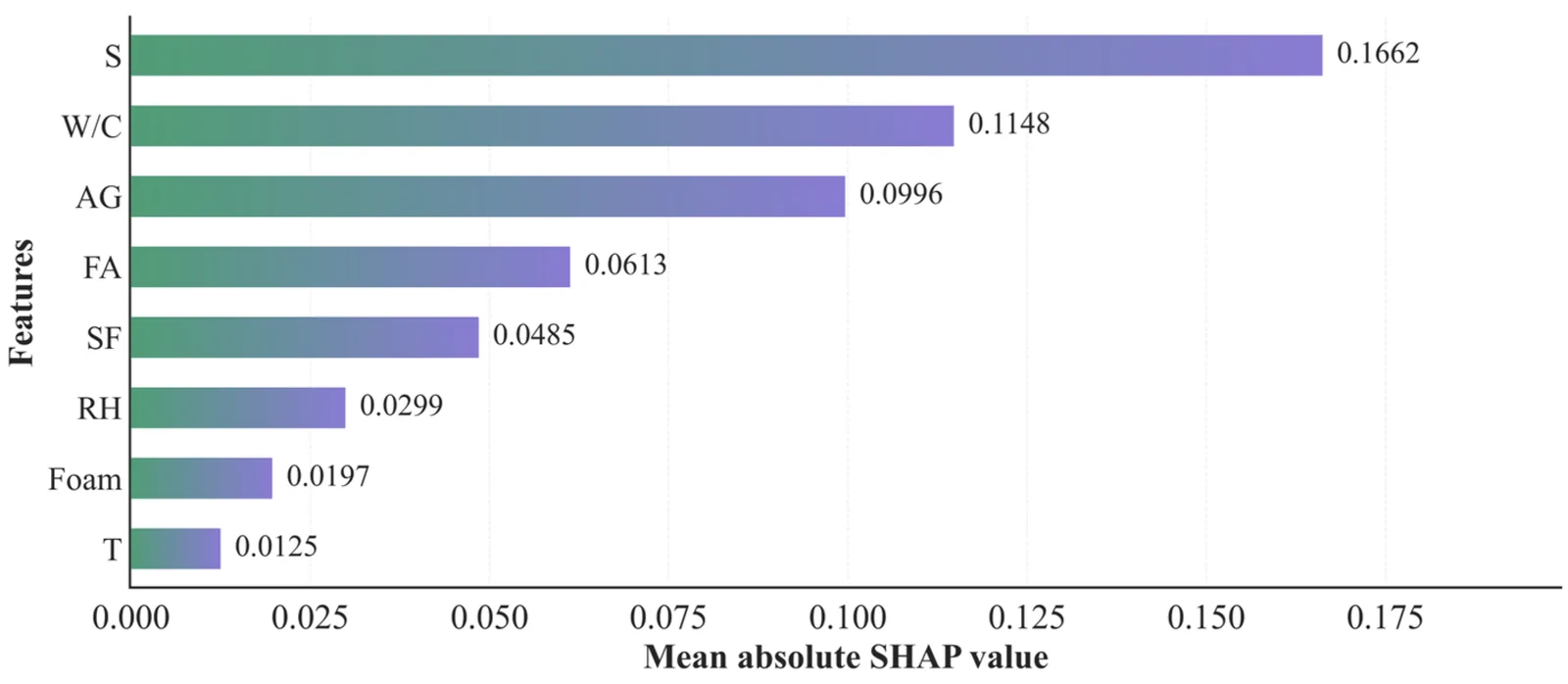
Global importance ranking of input variables for thermal conductivity prediction.

The mean absolute SHAP value ranking is shown in Figure 6, quantifying the global importance of each input variable. S exhibited the highest mean absolute SHAP value of 0.1662, followed by W/C and AG, with values of 0.1148 and 0.0996, respectively. This ranking indicates that sand content, water-to-cement ratio, and aerogel content made the largest contributions to thermal conductivity prediction. The mean absolute SHAP values of the remaining variables were all lower than 0.07, suggesting their relatively limited global contributions to the model output. These results further suggest that thermal conductivity prediction is not controlled by a single variable, but is jointly modulated by S, W/C, and AG. Specifically, sand content affects the continuity of the solid skeleton, W/C is closely associated with pore formation and matrix compactness, and aerogel introduces low–conductivity phases and modifies the pore structure.

**Figure 7:**
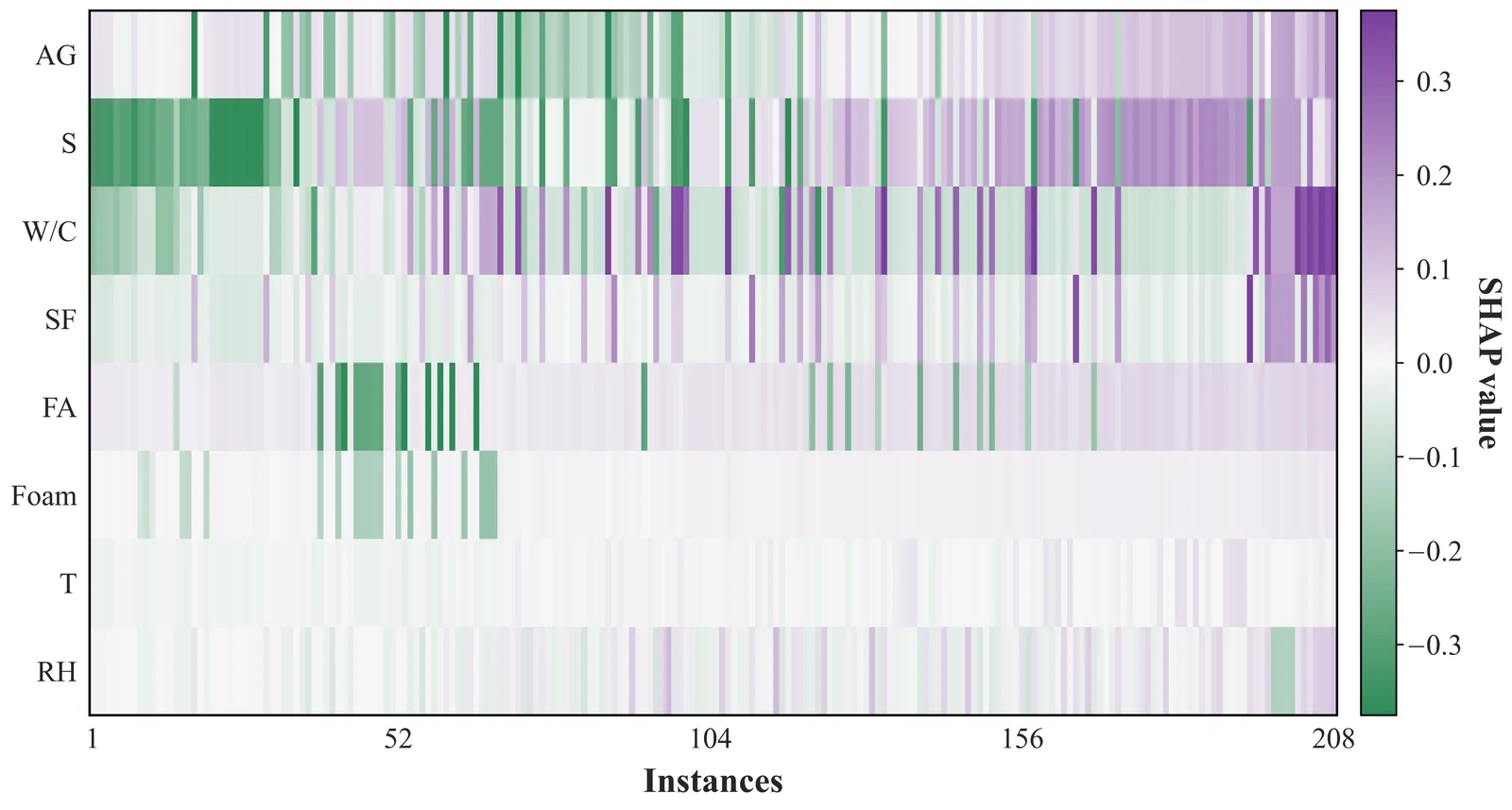
SHAP value heatmap of input variables for thermal conductivity prediction.

The sample–level SHAP contribution distributions of the input variables are shown in Figure 7. Pronounced sample-to-sample heterogeneity can be observed for S, W/C, and AG, which is consistent with their relatively high global importance shown in Figure 6. The SHAP values of S exhibited clear sign changes and magnitude variations across different samples, while AG and W/C also showed obvious variations in contribution direction and intensity depending on specific mix–proportion combinations. SF and FA showed moderate local contributions in some samples, whereas the SHAP values of Foam, T, and RH were mostly close to zero, suggesting relatively weak marginal contributions of these variables in most samples. These results reveal the sample heterogeneity and multivariable coupling characteristics of the thermal conductivity response of silica aerogel–incorporated cementitious composites, which are difficult to fully describe using linear correlation or single-factor analysis alone.

**Figure 8:**
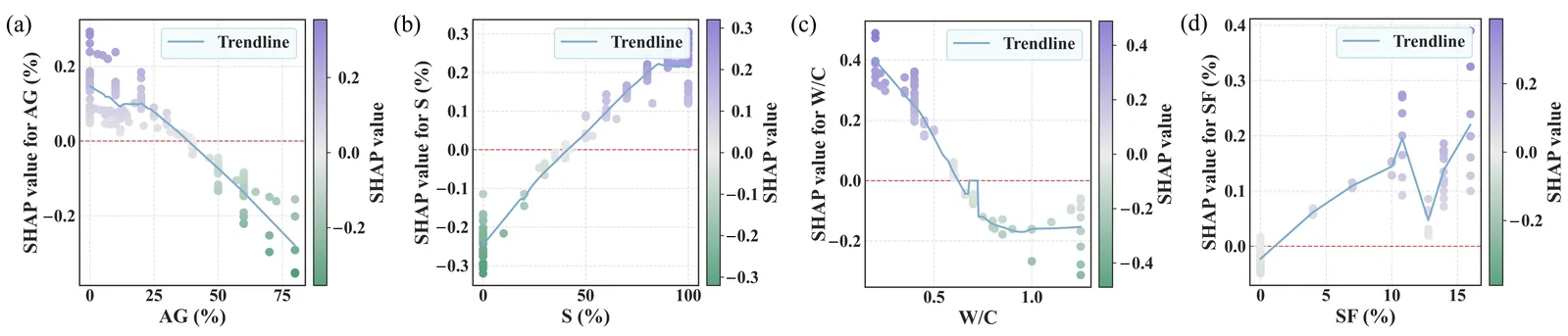

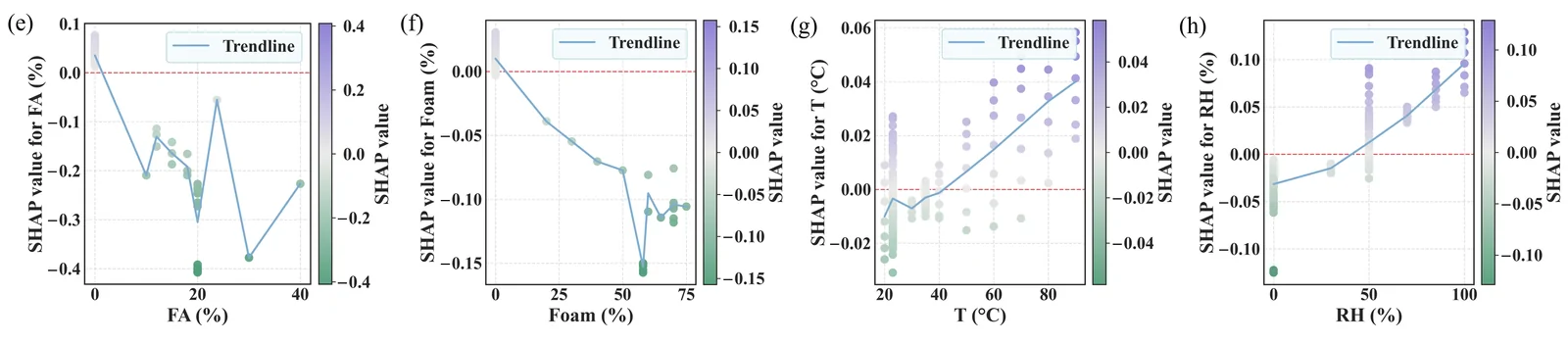
SHAP dependence plots of input variables for thermal conductivity prediction: (**a**) AG; (**b**) S; (**c**) W/C; (**d**) SF; (**e**) FA; (**f**) Foam; (**g**) T; (**h**) RH.

The relationships between input variables and their SHAP values are shown in Figure 8, revealing pronounced nonlinear contribution patterns of different variables to the predicted thermal conductivity. AG, S, and W/C exhibited relatively pronounced effects. As AG increased, the SHAP values gradually shifted from positive to negative, indicating that higher aerogel content tended to reduce the model-predicted thermal conductivity. When AG exceeded approximately 40%, its negative contribution became increasingly negative, suggesting that the reduction in predicted thermal conductivity became more pronounced at high aerogel contents. W/C also showed an overall decreasing trend, suggesting that higher W/C tended to decrease the predicted thermal conductivity. The SHAP values of S increased progressively from negative to positive as sand content increased, indicating that low S values reduced, whereas high S values increased, the predicted thermal conductivity. Foam and FA generally showed negative contributions within the current data domain, although their dependence patterns contained noticeable local fluctuations. SF exhibited an overall increasing positive contribution with increasing content, despite fluctuations at relatively high SF levels. T and RH showed smaller SHAP-value ranges than AG, S, and W/C, but both exhibited generally increasing trends. Higher T and RH values tended to produce positive contributions to the predicted thermal conductivity.

**Figure 9:**
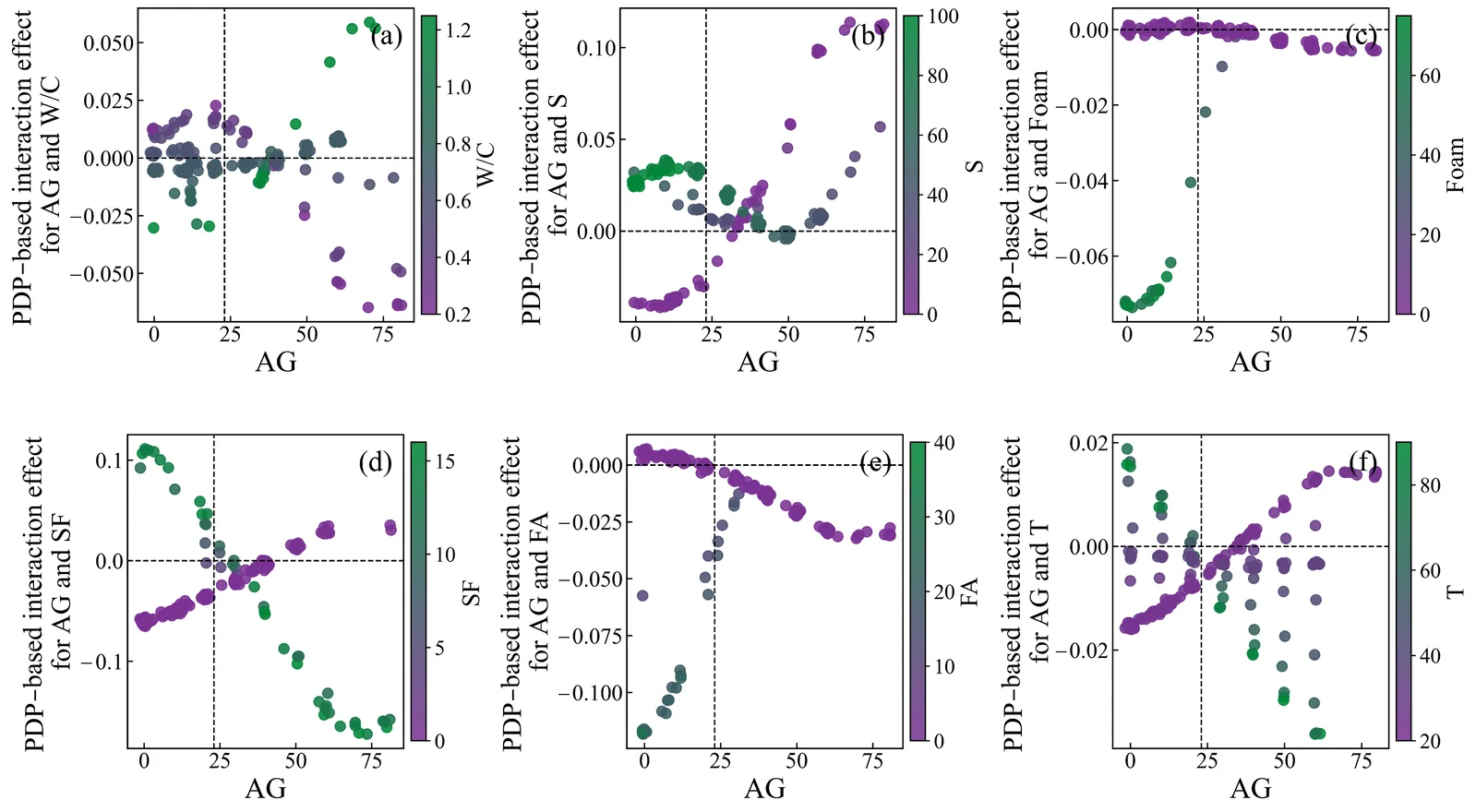
PDP-based pairwise interaction dependence plots of AG with selected input variables: (**a**) AG–W/C; (**b**) AG–S; (**c**) AG–Foam; (**d**) AG–SF; (**e**) AG–FA; (**f**) AG–T.

To further examine the second-order interrelations among input variables, PDP-based pairwise interaction dependence plots were constructed for AG and selected variables, as shown in Figure 9. The results indicate that the effect of AG on thermal conductivity is not completely independent, but varies with W/C, S, Foam, SF, FA, and T. Among these interactions, AG–W/C, AG–S, AG–SF, AG–FA, and AG–T exhibited evident nonlinear or sign-changing patterns, suggesting that the effect of aerogel on thermal conductivity is jointly modulated by matrix compactness, solid-skeleton composition, mineral admixtures, and testing temperature. By contrast, the AG–Foam interaction remained close to zero over most of the data range, although a pronounced negative interaction was observed when low AG was combined with high Foam content. This indicates a relatively weak overall interaction between AG and Foam, with a localized interaction under specific combinations. These findings further confirm that AG should be interpreted as an important variable acting within a multivariable mix-design and testing-condition domain, rather than as an isolated single-factor parameter.

### 2.6. Model Validation

**Figure 10:**
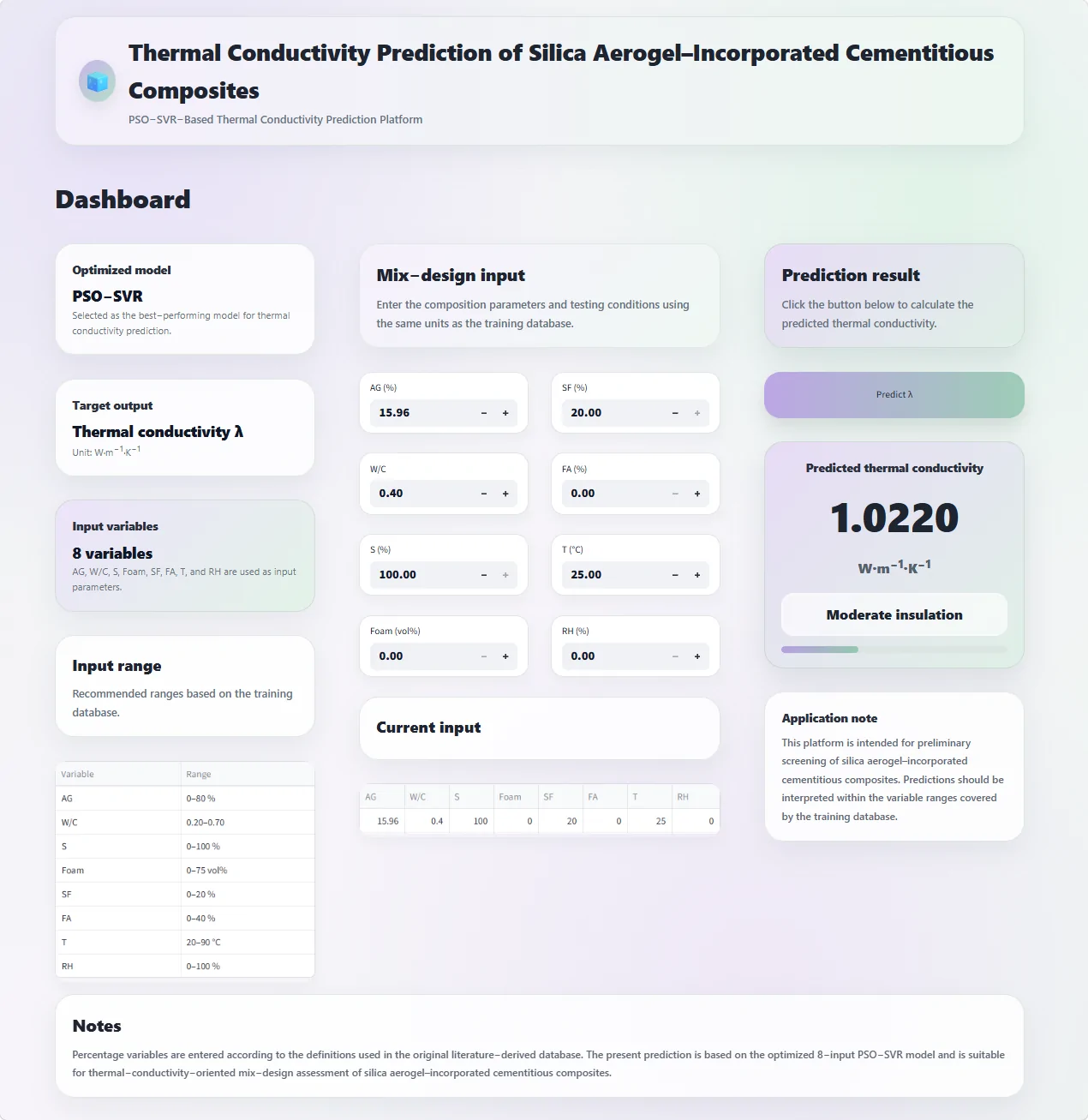
PSO–SVR-based thermal conductivity prediction platform.

Based on the optimized PSO–SVR model, a lightweight thermal conductivity prediction platform was developed, as shown in Figure 10. The platform uses the nine input variables adopted in this study, including AG, W/C, S, Foam, SF, FA, T, and RH, and directly outputs the predicted thermal conductivity of silica aerogel–incorporated cementitious composites. This platform provides a simple tool for rapid preliminary evaluation of mix-design parameters.

To further examine the external prediction capability of the optimized model, an external comparison was conducted using aerogel-incorporated cementitious composite data reported by Abbas et al. [26] and Han et al. [27]. The selected data records were converted into the same input format as the present database, and the measured and predicted thermal conductivity values are compared in Table 4.

As shown in Table 4, the PSO–SVR model generally captured the decreasing trend of thermal conductivity with increasing aerogel content. For the five external comparison records, the model achieved *R*^2^ = 0.9584, RMSE = 0.0618 W·m^−1^·K^−1^, and MAE = 0.0431 W·m^−1^·K^−1^. Although deviations were observed for some data points, the external comparison indicates that the proposed model has reasonable prediction capability for independent aerogel-incorporated cementitious composites.

### 2.7. Discussion

**Figure 11:**
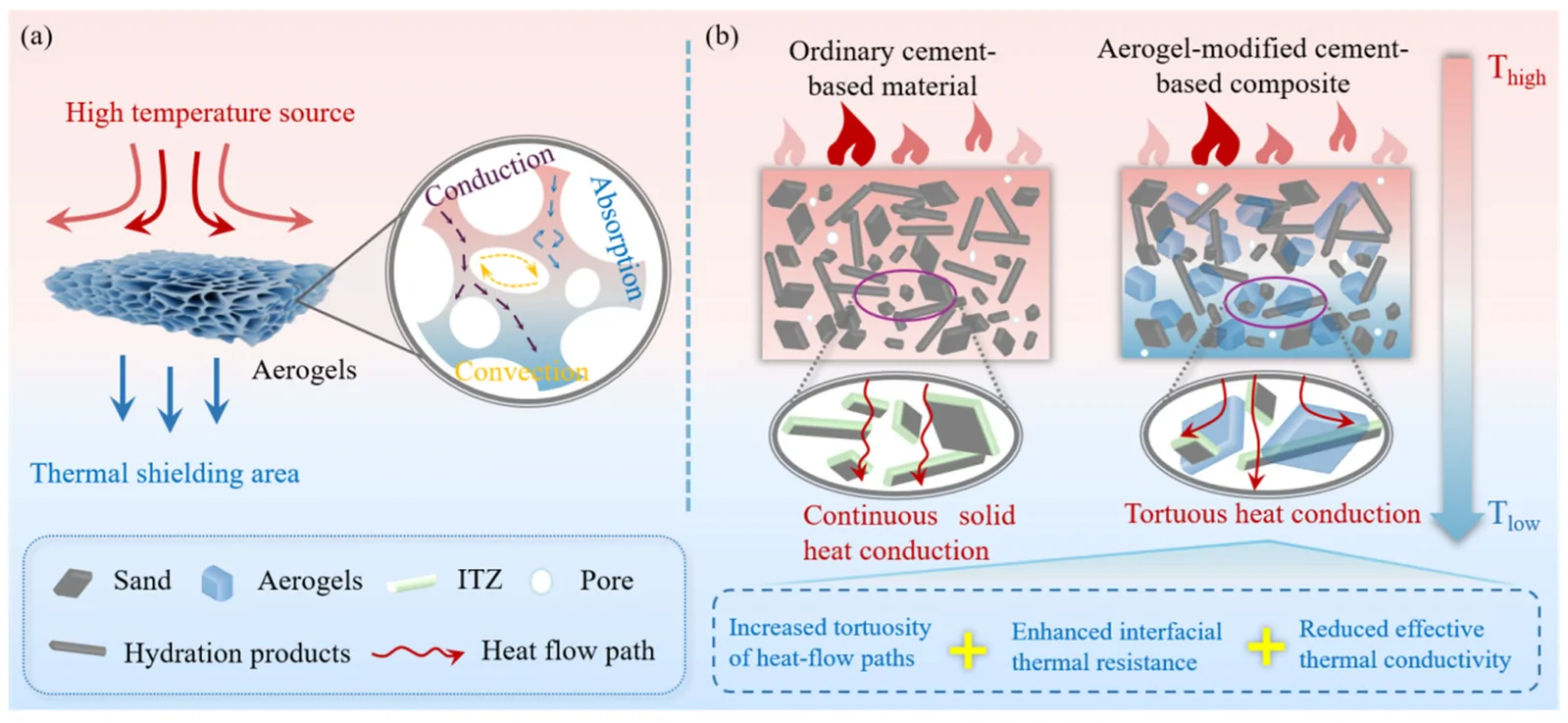
Schematic illustration of the thermal insulation mechanism of aerogel–modified cement–based composites: (**a**) thermal shielding effect; (**b**) heat-transfer path evolution.

The heat–transfer process in ordinary cementitious materials mainly depends on the solid skeleton composed of sand particles, hydration products, and hardened paste. When a relatively continuous contact network is formed among these solid components, heat can be transferred along low–thermal–resistance pathways, making solid–phase conduction dominant in the effective thermal conductivity. The evolution of heat–transfer pathways after aerogel incorporation is illustrated in Figure 11. Low–conductivity aerogel particles and the accompanying pore phases are embedded in the cementitious matrix, partially disrupting the original continuous solid heat–transfer network. As a result, heat flow is no longer transmitted through a single continuous skeleton, but is redistributed among the cementitious matrix, aerogel particles, pore phases, and interfacial transition zones (ITZs) [28,29]. Therefore, the regulation of effective thermal conductivity by aerogel is not governed solely by increased porosity, but is jointly controlled by the substitution of low-conductivity phases, the weakening of continuous solid heat-transfer pathways, increased tortuosity of heat-flow paths, and the accumulation of interfacial thermal resistance.

From the perspective of pore structure and interfacial heat transfer, the nanoporous structure and high specific surface area of aerogel help reduce the contribution of solid–phase heat transfer and restrict heat transfer through pore gas. For aerogel-containing cementitious composites, the suppression of gas-phase heat transfer is closely related to the Knudsen effect. When the pore size is comparable to or smaller than the mean free path of gas molecules, molecule–wall collisions become more frequent than molecule–molecule collisions, thereby reducing the effective thermal conductivity of the gas phase. Therefore, the nanoporous and mesoporous structures of silica aerogel can weaken gaseous heat conduction in the pore space and contribute to the overall thermal insulation effect. Previous studies have shown that aerogel can increase the number of micropores and mesopores in foamed cementitious materials and reduce their internal temperature response under high–temperature exposure [30]. Meanwhile, because aerogel and the cementitious matrix differ markedly in density, porosity, and thermal conductivity, additional interfacial thermal resistance is generated when heat flow crosses the aerogel–matrix interface. Relevant modeling studies have demonstrated that incorporating the ITZ effect into effective thermal conductivity prediction can significantly improve prediction accuracy, indicating that interfacial thermal resistance plays an important constraining role in multiphase heat–transfer processes [13]. This is consistent with the SHAP results in this study, where AG showed the highest contribution to thermal conductivity prediction, thereby providing mechanistic support for the key variable identified by the model. The positive contribution of S can be attributed to its enhancement of solid–skeleton continuity, whereas Foam and AG weaken continuous heat–transfer pathways by introducing low–conductivity porous phases.

Although the incorporation of aerogel can reduce thermal conductivity and thermal diffusivity, it may also increase porosity and water absorption and weaken mechanical properties [31,32,33]. Interactions between calcium silicate hydrate (C–S–H) and the aerogel network may also alter the pore structure and density. When the system becomes excessively densified, the contribution of solid-phase heat transfer may increase, thereby weakening its thermal-insulation advantage [34]. Therefore, the thermal design of silica aerogel–incorporated cementitious composites should account for the thermal-resistance enhancement associated with pore structure, the preservation of matrix continuity, and interfacial stability. From the perspective of geotechnical engineering applications, such low-thermal-conductivity cementitious materials can be used in underground-space envelopes, tunnel linings, thermal-insulation layers for foundations in cold regions, and thermal-regulation materials around ground-source heat pumps and energy piles. These applications can reduce heat exchange between the surrounding rock or soil and structures and improve the temperature stability of underground structures during service. Increasing aerogel content can weaken continuous heat-conduction pathways, but the associated increase in pore connectivity and reduction in mechanical performance may also limit engineering applicability.

Experimental results further indicate that this thermal–mechanical relationship strongly depends on aerogel dosage, particle characteristics, matrix composition, and interfacial conditions. Qu et al. [35] reported that incorporating 6% nano-SiO_2_ aerogel increased the 28-day flexural and compressive strengths of foamed concrete to 5.8 and 6.7 MPa, respectively. However, further increases in aerogel content resulted in strength deterioration because of particle agglomeration and weakened interfacial bonding. At an aerogel content of 10%, the thermal conductivity decreased to 0.186 W·m^−1^·K^−1^. By contrast, Kalkan and Gündüz [30] found that increasing the aerogel replacement level to 4.5 wt.% continuously reduced the compressive strength from 3.90 to 0.56 MPa, while the thermal conductivity decreased from 0.772 to 0.155 W·m^−1^·K^−1^. Marof and Siller [36] reported that increasing the aerogel content from 0 to 7 vol.% reduced the compressive strength from 9.6 to 7.8 MPa and the flexural strength from 2.31 to 1.80 MPa, while decreasing the thermal conductivity from 1.71 to 1.14 W·m^−1^·K^−1^. Their modified-aerogel–PET mortar retained compressive and flexural strengths of 6.3 and 1.54 MPa, respectively, at a thermal conductivity of 0.76 W·m^−1^·K^−1^. The statistical analysis of 85 aerogel-based renders by Ximenes et al. [37] also indicated that aerogel adversely affected compressive strength, whereas expanded clay made a positive contribution.

Therefore, in geotechnical engineering applications, the mix design of such materials should consider not only thermal conductivity but also volume stability and durability under groundwater exposure, freeze–thaw cycles, confining pressure, and long-term loading, to balance thermal-insulation efficiency, lightweight design, interfacial stability, and mechanical performance. Since the present model was developed using literature-derived data within the available variable ranges, differences in material systems and testing conditions may affect data consistency. Future studies should further expand the database by incorporating more standardized experimental records and microstructure-related descriptors to improve model reliability, generalization capability, and engineering applicability.

## 3. Conclusions

(1) In this study, a literature–derived database containing 208 data records was established for thermal conductivity prediction of silica aerogel–incorporated cementitious composites. XGBoost, RF, SVR, and their PSO–optimized models were developed and compared. Variable analysis indicated that thermal conductivity was jointly modulated by AG, S, W/C, Foam, SF, FA, T, and RH, rather than being controlled by a single factor. AG showed a strong negative correlation with thermal conductivity, whereas S exhibited a weak positive correlation, reflecting the combined effects of low–conductivity aerogel phases and solid–skeleton continuity on heat transfer.

(2) Model comparison showed that PSO optimization had a model-dependent influence on predictive performance, and its effectiveness varied with model structure and data distribution. Among the PSO-optimized models, PSO–SVR achieved the highest test-set *R*^2^ and the lowest RMSE, with values of 0.9306 and 0.1005, respectively. Its test-set MAE was 0.0658. Repeated random-split validation and external comparison further confirmed the stability and reasonable generalization capability of PSO–SVR. Overall, PSO–SVR achieved a good balance among prediction accuracy, residual stability, and generalization performance.

(3) SHAP analysis indicated that S was the most important variable, with a mean absolute SHAP value of 0.1662, followed by W/C and AG, with values of 0.1148 and 0.0996, respectively. Within the current data domain, these results suggest that thermal conductivity prediction is mainly governed by solid-skeleton continuity, pore-structure-related effects, and low-conductivity aerogel phases. Combined with the multiphase heat-transfer mechanism, silica aerogel reduces effective thermal conductivity mainly through the substitution of low-conductivity phases, the weakening of solid heat-transfer pathways, increased tortuosity of heat-flow paths, and the accumulation of interfacial thermal resistance.

Future work should further expand the database by integrating standardized experimental records, microstructural imaging data, and circular-economy-related indicators, with reference to recent advances in sustainable biopolymer and hydrogel materials [38,39]. Hybrid experimental–machine learning and physics-informed machine learning frameworks should also be developed to improve the reliability, interpretability, and practical applicability of thermal conductivity prediction.

## 4. Materials and Methods

### 4.1. Research Framework

The thermal conductivity of silica aerogel–incorporated cementitious composites is jointly controlled by multiple variables, including material composition, mix proportions, curing age, and environmental factors. Their heat–transfer process involves multiscale coupling among solid–phase heat transfer, pore structure, and interfacial thermal resistance, and the relationships among variables are usually highly nonlinear. Therefore, when describing thermal conductivity responses in multi–source datasets, traditional empirical models or single theoretical equations are often limited by variable coupling, sample heterogeneity, and the applicable ranges of model parameters. Machine learning methods can characterize the nonlinear mapping relationship between input variables and thermal conductivity without presupposing an explicit functional form, thereby providing a data–driven pathway for multivariable thermal conductivity response modeling.

**Figure 12:**
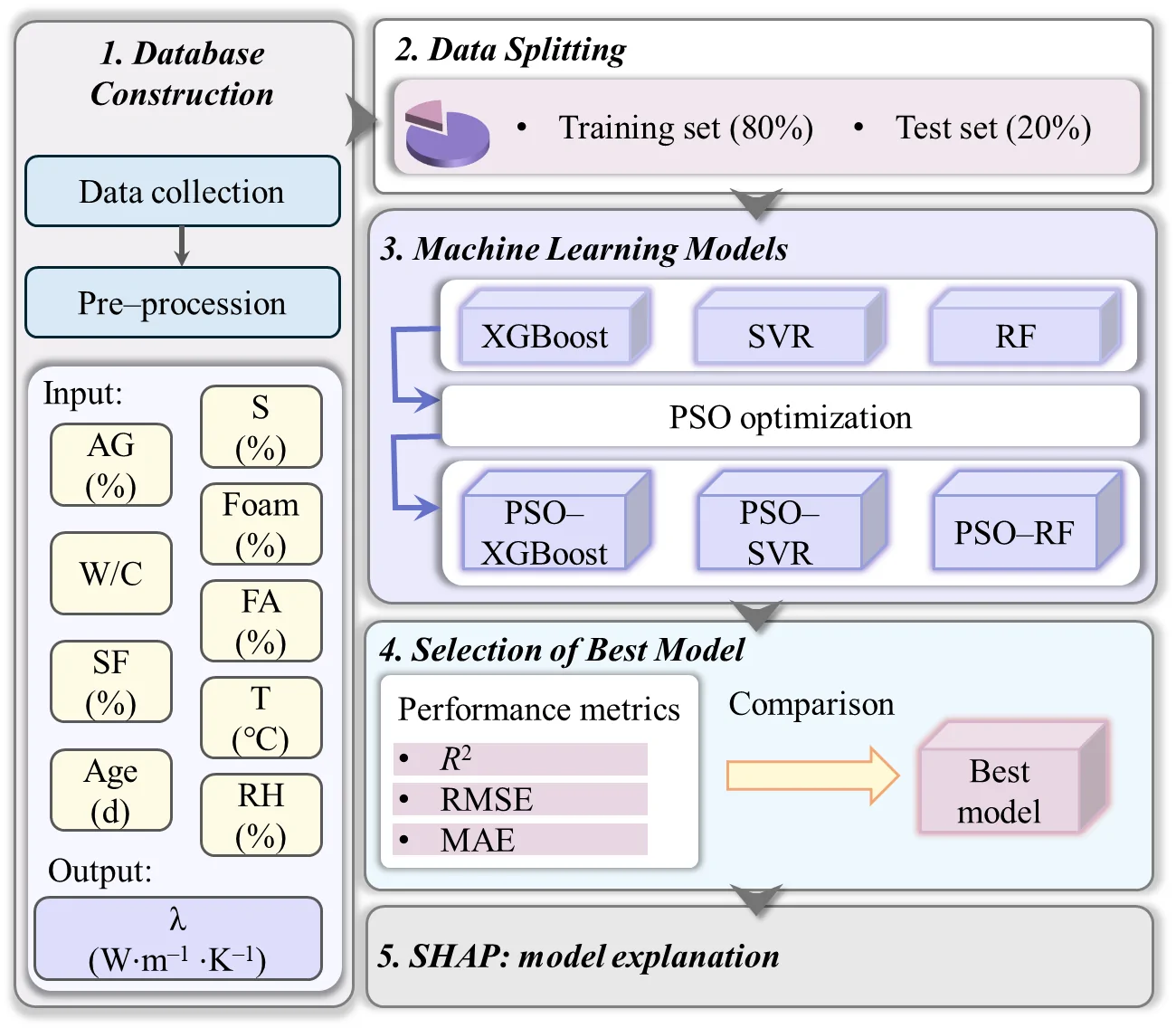
Machine learning framework for thermal conductivity prediction of silica aerogel–incorporated cementitious composites.

The technical route of this study is shown in Figure 12, and the data used were collected from published studies on silica aerogel–incorporated cementitious composites [7,8,9,12,13,14,30,35,36,37,40]. The literature sources used for database construction are summarized in Appendix A. During data collection, records containing mix–design parameters, material composition, testing conditions, and thermal conductivity values were preferentially selected. Data were extracted from the tables, figures, and textual descriptions reported in the original studies. When numerical values were presented graphically, they were obtained using a consistent digitization procedure and were cross-checked with the available textual or tabulated information where possible. To improve data consistency, variable names, units, and data formats from different literature sources were standardized before model development. Missing values in the database were supplemented according to the information provided in the original studies and the corresponding preprocessing rules. After data screening and standardization, a thermal conductivity database containing 208 valid data records was established. These procedures were used for data extraction, organization, and format standardization, and they do not affect the overall data structure or the main conclusions of this study. Silica aerogel content (AG), water–to–cement ratio (W/C), sand content (S), foam content (Foam), silica fume content (SF), fly ash content (FA), testing temperature (T), and testing relative humidity (RH) were used as input variables, while thermal conductivity (λ) was used as the output variable. The database was randomly divided into training and test sets at a ratio of 80:20 for model training and generalization performance evaluation.

During the model construction phase, Extreme Gradient Boosting (XGBoost), random forest (RF), and support vector regression (SVR) models were developed. Subsequently, particle swarm optimization (PSO) was used to perform global optimization of the model hyperparameters, leading to the development of PSO–XGBoost, PSO–RF, and PSO–SVR models. This approach aimed to mitigate the uncertainty inherent in empirical hyperparameter selection and enhance the models’ capacity to represent nonlinear thermal conductivity responses. Ultimately, the coefficient of determination (*R*^2^), root mean square error (RMSE), and mean absolute error (MAE) were employed to evaluate model performance comprehensively. Additionally, Shapley additive explanations (SHAP) were applied to assess the contribution magnitude and direction of each input variable to the predicted thermal conductivity.

### 4.2. Data Distribution and Correlation Analysis

**Figure 13:**
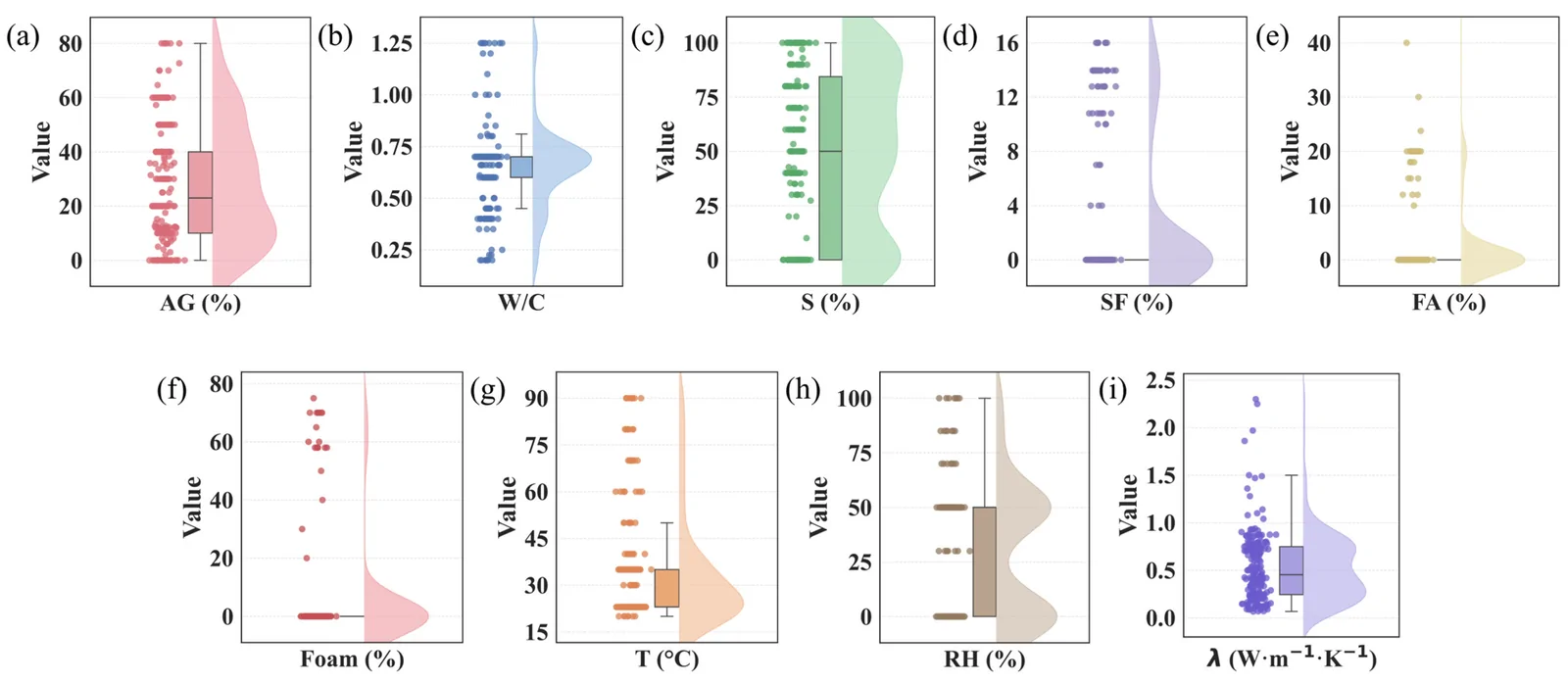
Data distributions: (**a**) AG; (**b**) W/C; (**c**) S; (**d**) SF; (**e**) FA; (**f**) Foam; (**g**) T; (**h**) RH; (**i**) λ.

The distribution characteristics of the input variables and thermal conductivity λ in the database are shown in Figure 13. Clear differences were observed among different variables in terms of value range, dispersion degree, and intervals with high sample concentrations, reflecting the strong heterogeneity of literature-derived data in material composition, mix design, and testing conditions. AG, S, RH, and λ exhibited relatively wide distribution ranges, reflecting the coverage of samples with different aerogel contents, sand contents, humidity conditions, and thermal conductivity levels. W/C was distributed within the range of approximately 0.20–1.25, with most samples concentrated around 0.50–0.75. T was mainly concentrated within the range of 20–30 °C, although several higher testing temperatures were also included. In contrast, SF, FA, and Foam were mainly distributed in low-content intervals, with a considerable proportion of samples having zero values, indicating differences in the value ranges of auxiliary components in the database. For the output variable, thermal conductivity λ showed a non–uniform distribution, with most samples concentrated in the low–thermal–conductivity range and only a few samples exhibiting relatively high thermal conductivity. This distribution pattern is consistent with the low–thermal–conductivity nature of silica aerogel–incorporated cementitious composites, while also indicating that λ can still vary considerably under different mix proportions and testing conditions.

**Figure 14:**
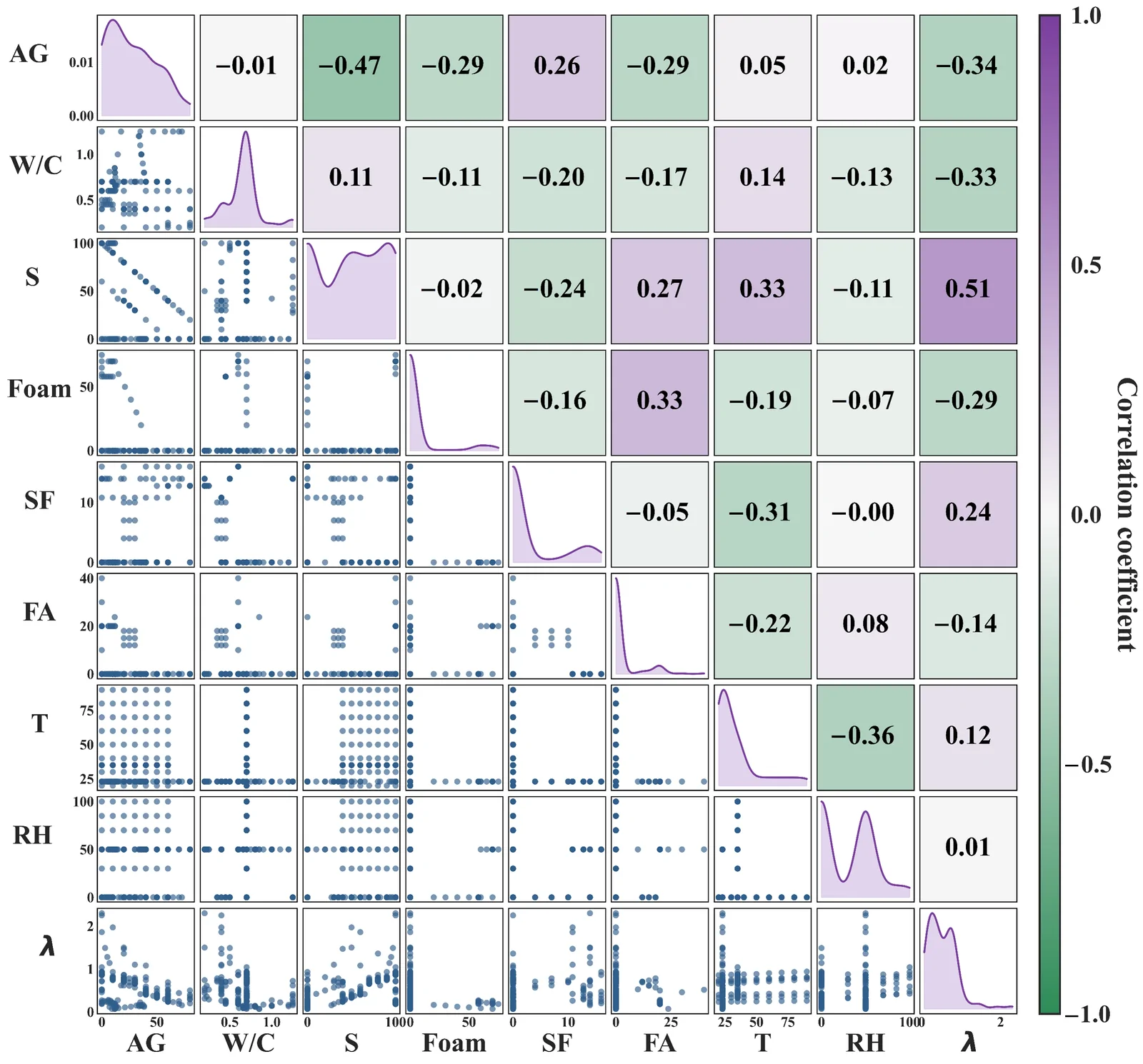
Correlation matrix and scatter distribution of input variables and thermal conductivity.

The correlations among the variables and between each variable and thermal conductivity λ are shown in Figure 14. AG demonstrated a significant negative correlation with λ, evidenced by a correlation coefficient of –0.34, which reflects the overall trend of decreasing thermal conductivity as aerogel content increases. This trend can be explained from the perspective of multiphase heat-transfer mechanisms: the low-density, high-porosity, and nanoporous structure of the aerogel phase disrupts continuous solid heat-transfer pathways, thereby enhancing thermal resistance at the interface between the aerogel and the cementitious matrix. W/C exhibited a negative correlation with λ, evidenced by a correlation coefficient of –0.33. This relationship may be attributed to the increased porosity and diminished matrix compactness associated with a higher W/C ratio. Conversely, S demonstrated a weak positive correlation with λ, with a correlation coefficient of 0.51, suggesting that an increase in sand content may improve the continuity of the solid skeleton, thereby enhancing heat transfer through solid-phase pathways. Foam exhibited a negative correlation with λ, evidenced by a correlation coefficient of –0.29, which highlights the adverse effect of foam pores on continuous heat-transfer pathways. In contrast, SF demonstrated a positive correlation with λ, with a correlation coefficient of 0.24. FA, however, displayed a weak negative correlation with λ, indicated by a correlation coefficient of –0.14. These findings suggest variations in the influence of different mineral admixtures on matrix densification, pore structure, and heat-transfer pathways. T showed a weak positive correlation with λ, with a correlation coefficient of 0.12, whereas RH was almost uncorrelated with λ, with a coefficient of 0.01. It should be noted that correlation analysis mainly reflects linear relationships among variables and is insufficient to reveal variable coupling and nonlinear effects. Therefore, machine learning models and SHAP analysis are still required to further interpret the conditional contributions of each variable to λ.

### 4.3. Machine Learning Models

This study employed Extreme Gradient Boosting (XGBoost), support vector regression (SVR), and random forest (RF) as the foundational machine learning models to establish the nonlinear mapping relationship between the input variables and thermal conductivity. These three models correspond to Boosting ensemble learning, kernel–based regression, and Bagging ensemble learning, respectively, and can characterize the variation patterns of thermal conductivity in silica aerogel–incorporated cementitious composites from different modeling mechanisms.

**Figure 15:**
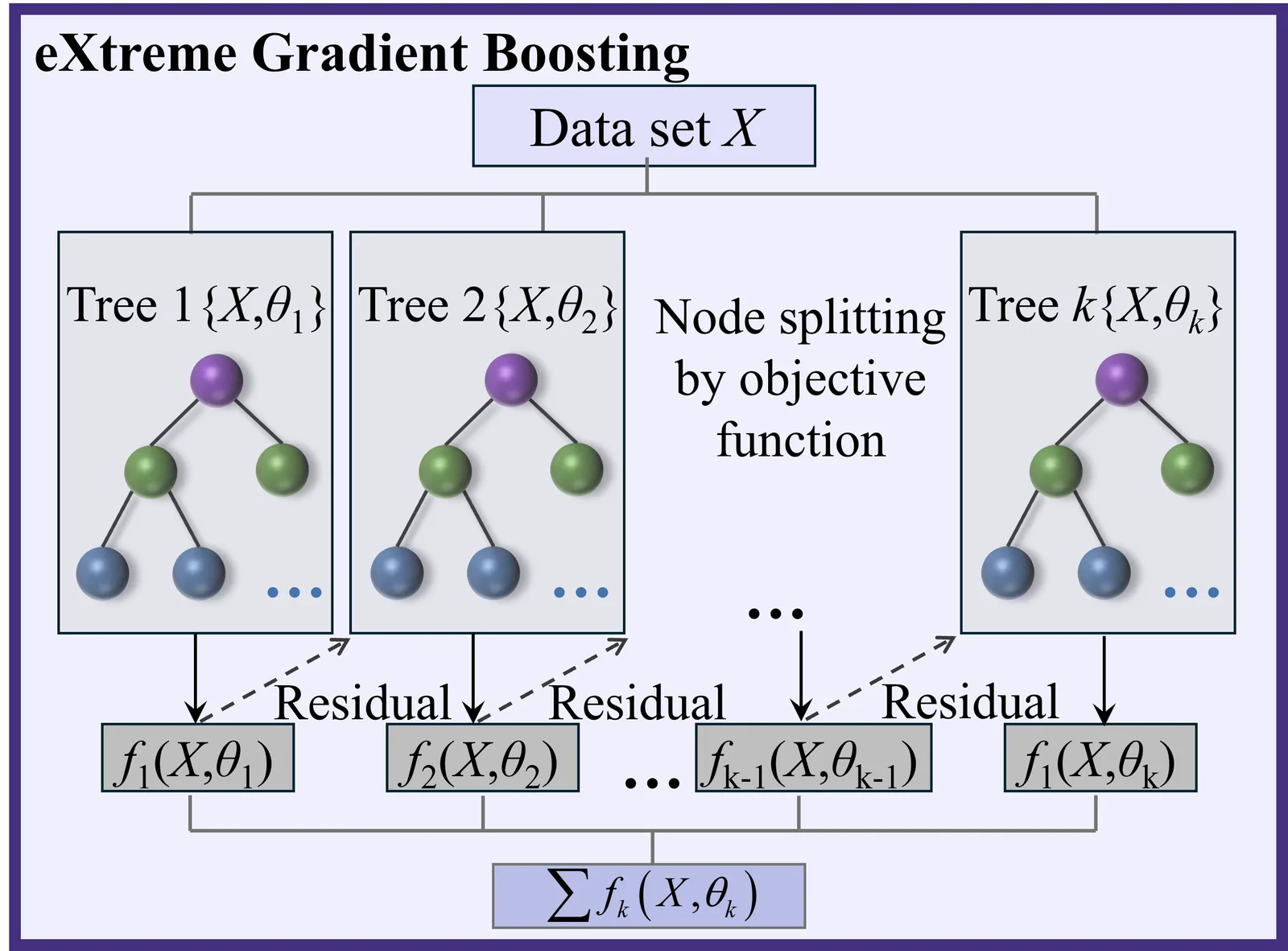
Schematic structure of the XGBoost model.

XGBoost is an ensemble learning model based on the gradient boosting framework [41], as illustrated in Figure 15. This model progressively constructs multiple regression trees, with subsequent trees iteratively correcting the residuals of the preceding model, thereby gradually reducing prediction errors. For a given sample $x_{i}$, the prediction of XGBoost can be expressed as the summation of the outputs of multiple regression trees:

(1) $${\hat{y}}_{i}=\sum_{k=1}^{K}f_{k}(x_{i}),\;f_{k}\in F$$

where ${\hat{y}}_{i}$ is the predicted value of the i sample, K is the number of regression trees, $f_{k}$ denotes the k regression tree, and F represents the function space of regression trees. The training process is achieved by minimizing an objective function consisting of a loss function and a regularization term:

(2) $$L=\sum_{i=1}^{n}l(y_{i},{\hat{y}}_{i})+\sum_{k=1}^{K}\Omega (f_{k})$$

where $l\left(y_{i},{\hat{y}}_{i}\right)$ is the loss function between the measured and predicted values, and $\Omega \left(f_{k}\right)$ is the regularization term of the k regression tree. In contrast to traditional gradient boosting trees, XGBoost incorporates regularization constraints into the objective function. This approach effectively manages tree–structure complexity, mitigates the risk of overfitting, and improves the model’s capacity to capture multivariable nonlinear relationships and interactions among features. Therefore, XGBoost is suitable for regression prediction problems involving multiple input variables, complex variable relationships, and pronounced nonlinear characteristics.

**Figure 16:**
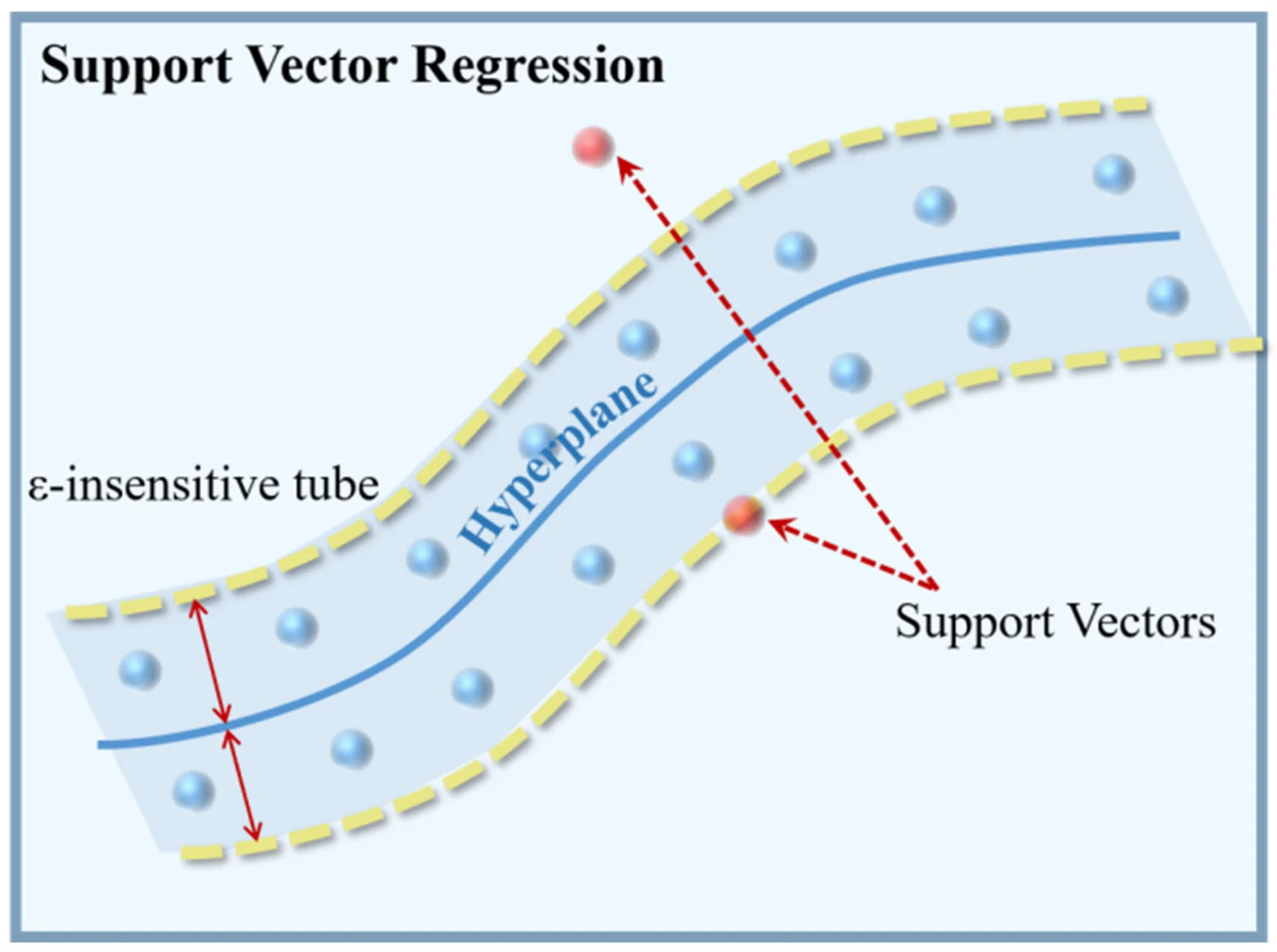
Schematic structure of the SVR model.

SVR extends the support vector machine (SVM) framework to address regression tasks [42], as illustrated in Figure 16. It maps input variables into a high–dimensional feature space using a kernel function and constructs an optimal regression function that operates within a specified error margin. The regression function can be expressed as:

(3) $$f(x)=\sum_{i=1}^{n}\left(\alpha_{i}-\alpha_{i}^{*})K(x_{i},x\right)+b$$

where f(x) is the SVR prediction function, $\alpha_{i}$ and $\alpha_{i}^{*}$ are Lagrange multipliers, $K\left(x_{i},x\right)$ is the kernel function, and b is the bias term. Unlike traditional regression methods that aim to minimize the overall error, SVR controls the error–tolerance interval using the ε–insensitive loss function, penalizes only the sample errors exceeding the ε–tolerance interval, and determines the main structure of the regression function mainly through support vectors. Therefore, SVR generally exhibits good generalization ability under limited–sample conditions. The study utilized the radial basis function (RBF) kernel to develop the support vector regression (SVR) model due to the significant nonlinear correlation observed between the thermal conductivity of cementitious composites containing silica aerogel and mix–proportion parameters. The RBF kernel is expressed as:

(4) $$K(x_{i},x_{j})=\exp(-\gamma \|x_{i}-x_{j}{\|}^{2})$$

where $x_{i}$ and $x_{j}$ denote input samples, and γ is the kernel parameter used to control the mapping range of samples in the high–dimensional feature space. The RBF kernel can enhance the ability of SVR to characterize complex nonlinear mapping relationships, making it suitable for predicting the thermal conductivity of silica aerogel–incorporated cementitious composites.

**Figure 17:**
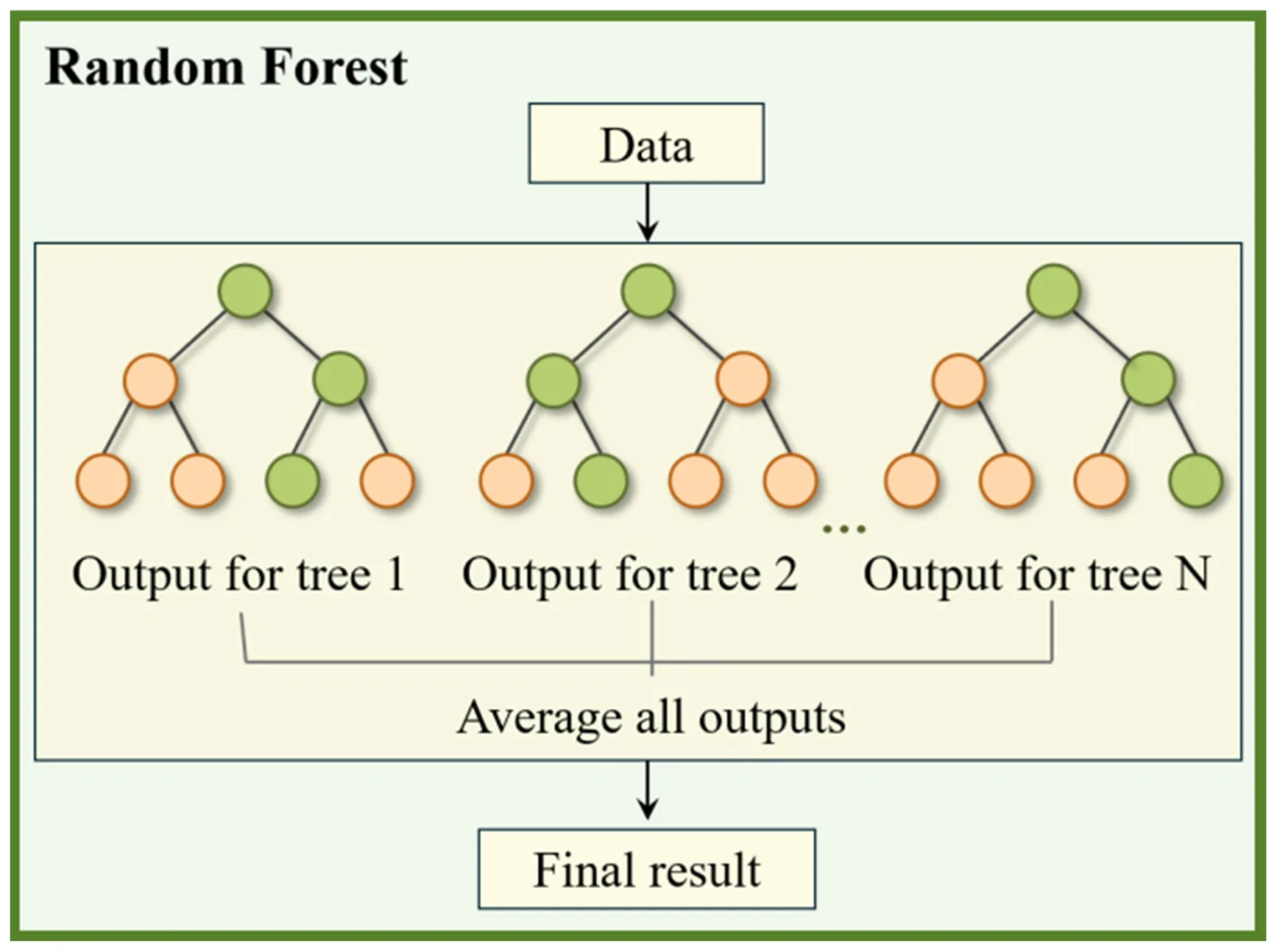
Schematic structure of the RF model.

Random Forest (RF) is an ensemble learning model that utilizes the Bagging technique [43], depicted in Figure 17. RF generates numerous regression trees via Bootstrap resampling and derives the ultimate prediction by averaging the outcomes of all trees. For a given input sample x, the prediction result of RF can be expressed as:

(5) $$\hat{y}=\frac{1}{B}\sum_{b=1}^{B}h_{b}(x)$$

where $\hat{y}$ is the predicted value of the model, B is the number of decision trees, and $h_{b}(x)$ represents the prediction result of the b decision tree for sample x. Since RF introduces both sample randomness and feature randomness during tree construction, it can reduce the sensitivity of a single decision tree to local sample structures, thereby improving the stability of model prediction.

Considering that the prediction performance of machine learning models is highly sensitive to hyperparameter settings, particle swarm optimization (PSO) was further employed in this study to perform global optimization of the key hyperparameters of XGBoost, SVR, and RF. Accordingly, PSO–XGBoost, PSO–SVR, and PSO–RF models were constructed. To verify the reliability of the optimization process, five-fold cross-validation was used as the fitness evaluation strategy during PSO optimization. The hyperparameter search ranges were determined based on relevant machine learning studies on cementitious material property prediction and preliminary trial calculations. The final optimized hyperparameters are summarized in Table 1. PSO simulates the cooperative search process of particle swarms in the search space and continuously updates the velocity and position of each particle, allowing candidate hyperparameter combinations to gradually converge toward a globally favorable region. The velocity and position updating processes of particles can be expressed as:

(6) $$v_{i}^{t+1}=\omega v_{i}^{t}+c_{1}r_{1}(p_{i}^{t}-x_{i}^{t})+c_{2}r_{2}(g^{t}-x_{i}^{t})$$

(7) $$x_{i}^{t+1}=x_{i}^{t}+v_{i}^{t+1}$$

where $x_{i}^{t}$ and $v_{i}^{t}$ denote the position and velocity of the i particle at the t iteration, respectively; $p_{i}^{t}$ is the historical best position of the individual particle; $g^{t}$ is the current global best position of the particle swarm; ω is the inertia weight; $c_{1}$ and $c_{2}$ are learning factors; and $r_{1}$ and $r_{2}$ are random numbers between 0 and 1. Through the above iterative updating process, PSO can reduce the subjectivity associated with empirical hyperparameter tuning and enable each model to obtain a relatively reasonable hyperparameter combination under a unified parameter–search framework.

All machine learning analyses were implemented in Python 3.12.4 under the Anaconda environment. The main packages used in this study included NumPy 2.2.6, pandas 3.0.2, scikit-learn 1.8.0, XGBoost 3.2.0, SHAP 0.51.0, and matplotlib 3.10.8. The random seed was fixed at 42 to improve the reproducibility of data partitioning and model training. During PSO optimization, the population size and maximum number of iterations were set to 20 and 30, respectively. The inertia weight was set to 0.8, and the cognitive and social acceleration coefficients were both set to 0.5. The PSO process was terminated when the maximum number of iterations was reached.

The prediction performance of each model was assessed using the coefficient of determination (*R*^2^), root mean square error (RMSE), and mean absolute error (MAE) as evaluation metrics in this study. Their calculation formulas are as follows:

(8) $$R^{2}=1-\frac{\sum_{i=1}^{n}{\left(y_{i}-{\hat{y}}_{i}\right)}^{2}}{\sum_{i=1}^{n}{\left(y_{i}-\bar{y}\right)}^{2}}$$

(9) $$\mathrm{RMSE}=\sqrt{\frac{1}{n}{\sum_{i=1}^{n}\left(y_{i}-{\hat{y}}_{i}\right)}^{2}}$$

(10) $$\mathrm{MAE}=\frac{1}{n}\sum_{i=1}^{n}\left|y_{i}-{\hat{y}}_{i}\right|$$

where $y_{i}$ is the measured value, ${\hat{y}}_{i}$ is the predicted value, $\bar{y}$ is the mean value of the measured values, and n is the number of samples. R^2^ reflects the ability of the model to explain the variation in the target variable, and a value closer to 1 indicates better fitting performance. RMSE is particularly sensitive to significant prediction errors, providing insight into the overall spread of prediction errors. On the other hand, MAE represents the average absolute deviation between predicted and actual values. By utilizing these three metrics together, one can assess model performance comprehensively, considering explanatory power, error distribution, and average deviation, thus mitigating the bias that may arise from relying on a single metric.

## Figures and Tables

**Table 1:** Optimized hyperparameter configurations of the PSO–optimized models.

Model	Hyper–Parameter	Optimized Value
PSO–XGBoost	n_estimators	300.000
	max_depth	3.000
	learning_rate	0.120
	subsample	0.717
	colsample_bytree	0.900
	reg_alpha	1.546
	reg_lambda	4.977
PSO–RF	n_estimators	50.000
	max_depth	7.000
	min_samples_split	4.000
	min_samples_leaf	1.000
	max_features	0.618
PSO–SVR	kernel	rbf
	C	1.832
	epsilon	0.077
	gamma	0.063

**Table 2:** Comparative prediction performance of different machine learning models.

Model	Train			Test		
	*R* ^2^	RMSE	MAE	*R* ^2^	RMSE	MAE
XGBoost	0.9999	0.0033	0.0020	0.9379	0.0995	0.0617
SVR	0.9107	0.1091	0.0717	0.8689	0.1446	0.0865
RF	0.9807	0.0507	0.0231	0.9110	0.1191	0.0672
PSO–XGBoost	0.9963	0.0223	0.0165	0.9302	0.1055	0.0561
PSO–SVR	0.9206	0.1028	0.0608	0.9306	0.1005	0.0658
PSO–RF	0.9557	0.0768	0.0410	0.8949	0.1294	0.0803

**Table 3:** Repeated random-split validation results of the optimized models.

Model	*R* ^2^	RMSE	MAE
PSO–XGBoost	0.9010 ± 0.0600	0.1130 ± 0.0508	0.0593 ± 0.0217
PSO–RF	0.8468 ± 0.0914	0.1412 ± 0.0670	0.0751 ± 0.0280
PSO–SVR	0.9051 ± 0.0472	0.1098 ± 0.0390	0.0711 ± 0.0151

**Table 4:** External comparison between measured and predicted thermal conductivity values.

Sample	AG/%	W/C	S/%	Foam/vol%	SF/%	FA/%	T/°C	RH/%	λ/(W·m^−1^·K^−1^)	
									Test	Predicted
Comparison 1	15.96	0.40	100.00	0.00	20.00	0.00	25.00	0.00	1.14	1.0220
Comparison 2	31.93	0.40	75.00	0.00	20.00	0.00	25.00	0.00	0.80	0.8696
Comparison 3	46.75	0.40	50.00	0.00	20.00	0.00	25.00	0.00	0.62	0.6173
Comparison 4	62.34	0.40	25.00	0.00	20.00	0.00	25.00	0.00	0.33	0.3450
Comparison 5	30.00	0.385	70.00	0.00	10.00	0.00	23.00	0.00	1.10	1.1103

## Data Availability

Data are contained within the article.

## Appendix A. List of References for Database

**Ref.**	**Data Source**	**Material System**	**No. of Records**
[7]	Wang et al., 2019	Aerogel-incorporated concrete under hygrothermal environments	98
[8]	Ng et al., 2015	Aerogel-incorporated ultra-high-performance concrete/mortar	18
[9]	Rong et al., 2023	Alkali-excited fly ash aerogel foam concrete	17
[12]	Li et al., 2019	Ultra-light thermal-insulative aerogel foam concrete	6
[13]	Li et al., 2025	AICC effective thermal conductivity with ITZ effects	9
[14]	Xiong et al., 2025	Dual-optimized aerogel concrete	9
[30]	Kalkan et al., 2023	Silica-aerogel-added lightweight cement-based composite mortar	8
[35]	Qu et al., 2025	Nano-SiO_2_-aerogel foamed concrete	6
[36]	Marof and Siller, 2025	Silica-aerogel- and recycled-PET-incorporated cement mortar	4
[37]	Ximenes et al., 2016	Aerogel-based rendering mortar	26
[40]	Gao et al., 2014	Aerogel-incorporated concrete	7
